# Apical proximal tubule fatty acid uptake–generated ceramides cause endoplasmic reticulum stress from altered membrane fluidity

**DOI:** 10.1172/jci.insight.199699

**Published:** 2026-06-18

**Authors:** Zhiyu Liu, Robert J. Gaivin, Shenaz Khan, Vincent Li, Amal Chaba, Fraser J. Moss, Usman Sabir, Takhar Kasumov, Tingwei Mu, Jeffrey R. Schelling

**Affiliations:** 1 Department of Physiology and Biophysics, Case Western Reserve University, Cleveland, Ohio, USA.; 2 Department of Pharmaceutical Sciences, Northeast Ohio Medical University, Rootstown, Ohio, USA.; 3 Department of Medicine, University Hospitals Cleveland Medical Center, Cleveland, Ohio, USA.

**Keywords:** Cell biology, Nephrology, Chronic kidney disease

## Abstract

Circulating fatty acids (FAs) are constitutively taken up by basolateral kidney proximal tubule transporters and are the preferred metabolic substrate. In many chronic kidney diseases, the damaged glomerular filtration barrier permits passage of albumin-bound FAs, which are reabsorbed by apical FA transport protein-2 (FATP2). Bilateral FA uptake leads to lipotoxicity and progressive renal function decline, but the relative apical versus basolateral contribution and intracellular mechanisms are not established. Apical or bilateral (but not basolateral) palmitate incubation with human proximal tubule cells stimulated endoplasmic reticulum (ER) stress gene expression, ER stress pathway activation, and ER fragmentation. Apical or bilateral palmitate was associated with reduced lipid droplets and decreased expression of ER-localized lipid droplet biogenesis transcripts. Inhibition of lipid droplet formation also precipitated ER stress, suggesting diminished sequestration of FA metabolites as the cause. Indeed, C16:0 ceramide was increased in bilateral palmitate-treated cells and in kidneys from mice that phenocopy progressive diabetic kidney disease. Ceramide synthesis inhibition abrogated ER stress, and transfection with C16:0 ceramide decreased ER membrane fluidity and caused ER stress. We conclude that aberrant filtration and uptake of FAs by apical FATP2 exceeded the capacity for lipid droplet incorporation and led to cytotoxicity from ceramide-induced ER lipid bilayer stress.

## Abstract 2

Diabetic kidney disease is associated with fat accumulation in kidney cells. This paper describes one mechanism by which the fats are toxic.

## Graphical abstract

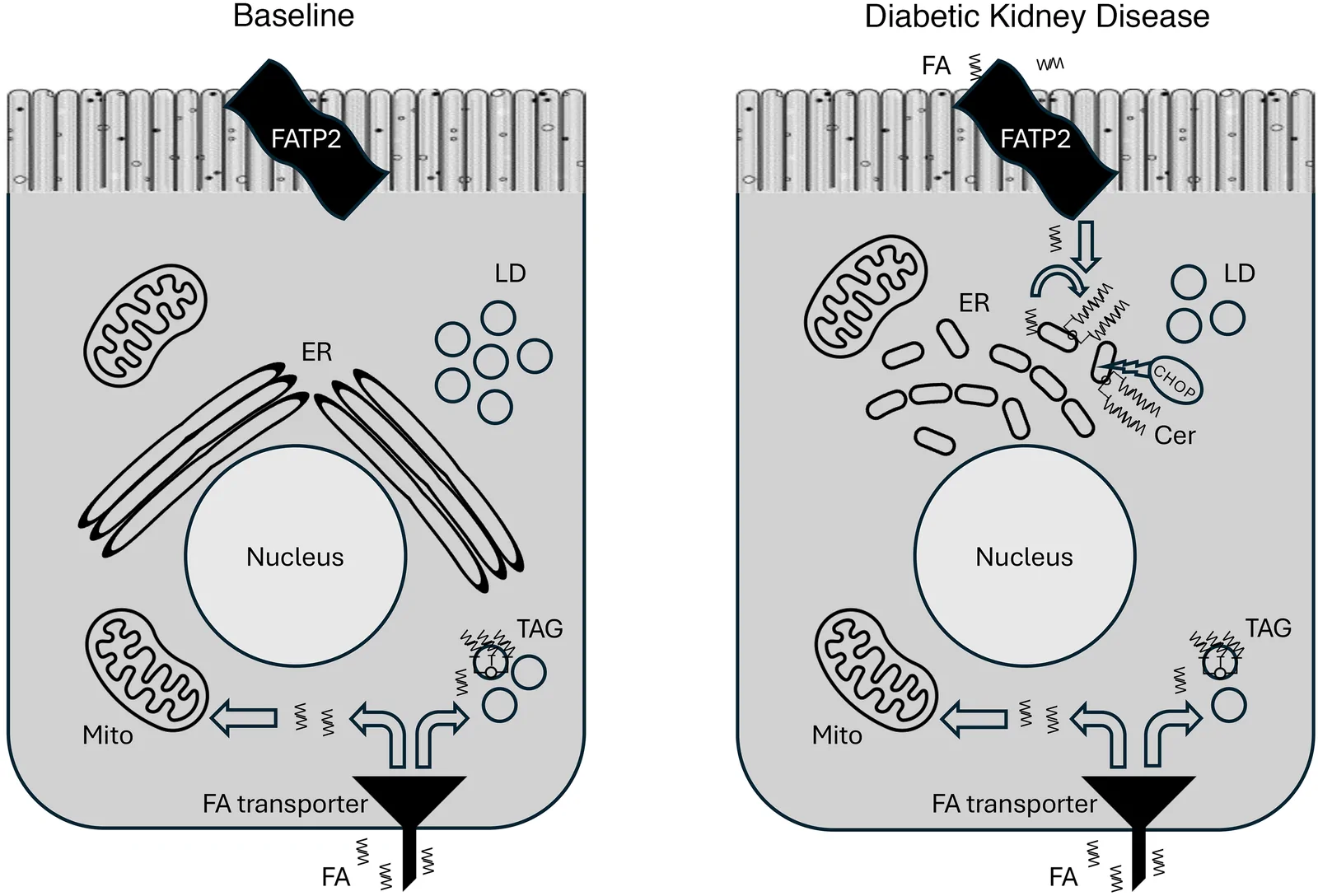

## Introduction

The US prevalence of diabetes in 2023 was 40 million (1), and it represents a major public health problem owing to morbidity and mortality associated with end-organ complications. A devastating complication is diabetic kidney disease (DKD), which is overwhelmingly the most common cause of end-stage kidney disease (2) and is treatable only with dialysis or kidney transplantation. Blood pressure and glucose control are important interventions to slow DKD progression, but neither is sufficient to extinguish diabetic complications (3).

DKD is commonly classified as a glomerular disease, which is reflected by albuminuria as a biomarker of early progression. However, tubular atrophy is superior to glomerular pathology as a predictor of non-diabetic and diabetic kidney disease progression (4–7), which may drive glomerular pathology and dysfunction (8, 9). Of many possible causes of tubular atrophy, extensive evidence exists for tubular lipotoxicity (10–18). Circulating fatty acids (FAs) are constitutively taken up by basolateral (BL) proximal tubule transporters and are the preferred metabolic substrate (19). In DKD, the damaged glomerular filtration barrier permits passage of albumin-bound FAs, which are reabsorbed by apical (AP) FA transport protein-2 (FATP2) (20–22). The resulting bilateral FA uptake leads to lipotoxicity and progressive renal function decline. In addition to increased FA uptake (12, 20–25), enhanced FA synthesis (13, 15) and decreased catabolism by β-oxidation (11, 15, 18) have also been implicated in tubular atrophy pathogenesis.

Unmetabolized, intracellular FAs are compartmentalized as triacylglycerol in lipid droplets (LDs), which are single-phospholipid-membrane organelles that buffer against the damaging effects of excess FA metabolites. Of long-chain FAs, palmitate (C16:0) is problematic because (a) circulating palmitate concentrations are increased with diabetes (26), and (b) palmitate does not readily incorporate into triacylglycerol and LDs (27–29), and is instead shunted to ceramide or diacylglycerol formation in the endoplasmic reticulum (ER) or leads to stimulation of reactive oxygen species generation and apoptosis (30–34). In vivo, diabetic mice fed a high-fat diet (HFD) enriched for unsaturated FAs exhibited increased LDs and less proximal tubule cytotoxicity compared with diabetic mice on a saturated high-fat diet (29). However, the relative AP versus BL uptake, and effects on glomerular filtration rate (GFR), were not assessed.

The fate of unsequestered FAs in proximal tubule cells in the context of DKD is not established. Proximal tubule uptake of palmitate could lead to cytotoxic C16:0 ceramide formation, since palmitate is a key precursor for both the sphinganine backbone and the C16:0 ceramide acyl chain addition. However, lipidomic studies have yielded conflicting results regarding tissue ceramide concentrations in mouse models of DKD (35–37). Furthermore, the role of ceramides as cause or effect of ER stress is ambiguous and perhaps conditional. Ceramides are synthesized in the ER and may therefore be increased as a consequence of ER stress (38). Alternatively, excess saturated FA metabolites, including ceramides, can be inserted into ER membranes, which reduces membrane fluidity, thereby contributing to lipid bilayer stress and proximity-induced activation of the ER stress enzymes inositol-requiring enzyme 1 (IRE1) and protein kinase R–like ER kinase (PERK) (34–42). Whether ceramides specifically cause ER stress through membrane phase transition and lipid bilayer stress has not been fully investigated.

In this report, we aimed to clarify several issues regarding mechanisms of proximal tubule lipotoxicity: (a) the relative contributions of AP versus BL FA uptake, (b) the roles of ER and LDs, and (c) whether C16:0 ceramide is increased, and if so, how it contributes to cytotoxicity.

## Results

### Conditional proximal tubule FATP2 deletion ameliorates DKD induced by HFD plus streptozotocin.

**Figure 1:**
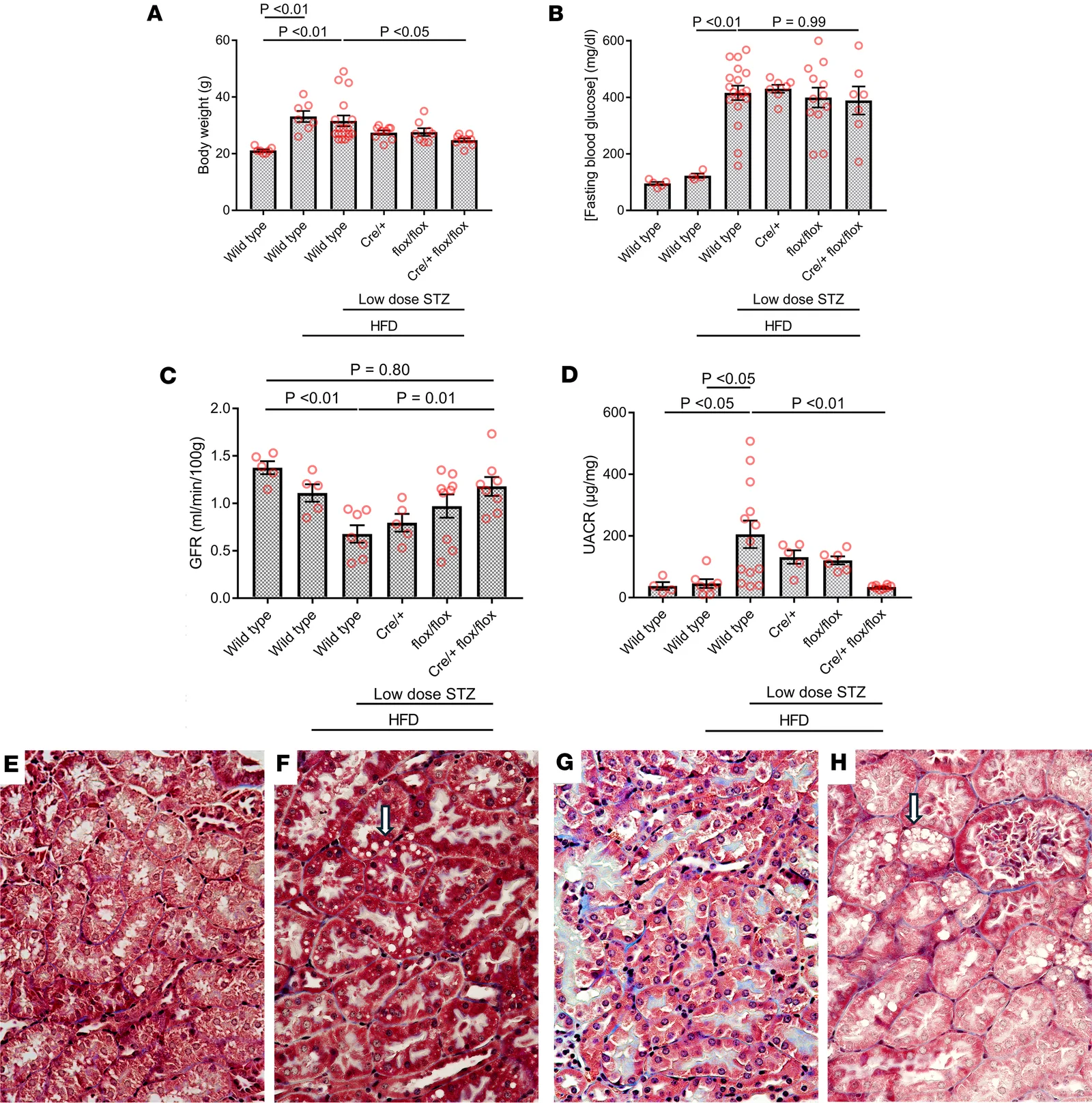
Conditional proximal tubule FATP2 deletion ameliorates HFD + STZ–induced DKD. (**A**–**D**) Conditional proximal tubule FATP2 deletion (GGT1-Cre^+/–^ *Slc27a2^fl/fl^*) and genotype control mice were generated as described in Methods. At 6 weeks of age, mice were placed on a high-fat diet (HFD) for 6 months and treated with low-dose streptozotocin (STZ) at 3 months to phenocopy kidney disease from type 2 diabetes. At the end of the HFD period, body weight (**A**), fasting blood glucose (**B**), glomerular filtration rate (GFR; **C**) and urine albumin/creatinine ratio (UACR; **D**) were measured, as described in Methods. Each symbol represents a value from an individual mouse at the end of the 6-month protocol. Histograms represent mean ± SEM, and data were analyzed by 1-way ANOVA and Tukey’s post hoc test for multiple comparisons. (**E**–**H**) Representative histology sections from wild-type (**E**), wild-type + HFD/STZ (**F**), *Slc27a2^fl/fl^* + HFD/STZ (**G**), and GGT1-Cre^+/–^ *Slc27a2^fl/fl^* + HFD/STZ (**H**) kidney cortex were stained with Masson’s trichrome, as described in Methods. Arrows demarcate proximal tubule cytoplasmic vacuoles. (**E**-**H**) Original magnification, ×400.

In mouse models of DKD, global *Slc27a2* deletion rescued GFR (22). To determine whether the effect was due to kidney FATP2, mice with conditional proximal tubule knockout were created (42). Streptozotocin-treated (STZ-treated), HFD-fed mice with *Slc27a2* deleted weighed less than wild-type mice (Figure 1A). Figure 1B demonstrates that conditional *Slc27a2* deletion did not affect fasting blood glucose concentrations, consistent with published results (42). Wild-type, Cre, and floxed control mice treated with HFD plus low-dose STZ developed GFR decline, consistent with previous reports (22). Figure 1C shows that proximal tubule *Slc27a2* deletion rescued GFR to near baseline levels in HFD + STZ mice. Albuminuria was also reduced in HFD + STZ mice with conditional *Slc27a2* knockout (Figure 1D). The most striking pathologic finding in HFD + STZ kidneys was proximal tubule cytoplasmic vacuoles (presumed lipid droplets; Figure 1, E–H), which were increased in *Slc27a2-*knockout mice on HFD + STZ (Figure 1H). Because (a) GFR improved in conditional-*Slc27a2*-KO mice, (b) FATP2 is the predominant proximal tubule FA transporter (21), and (c) within the kidney, FATP2 is expressed exclusively in the AP proximal tubule membrane (21), we conclude that AP FA uptake contributes to DKD pathogenesis.

### ER stress is induced by AP proximal tubule palmitate exposure.

**Figure 2:**
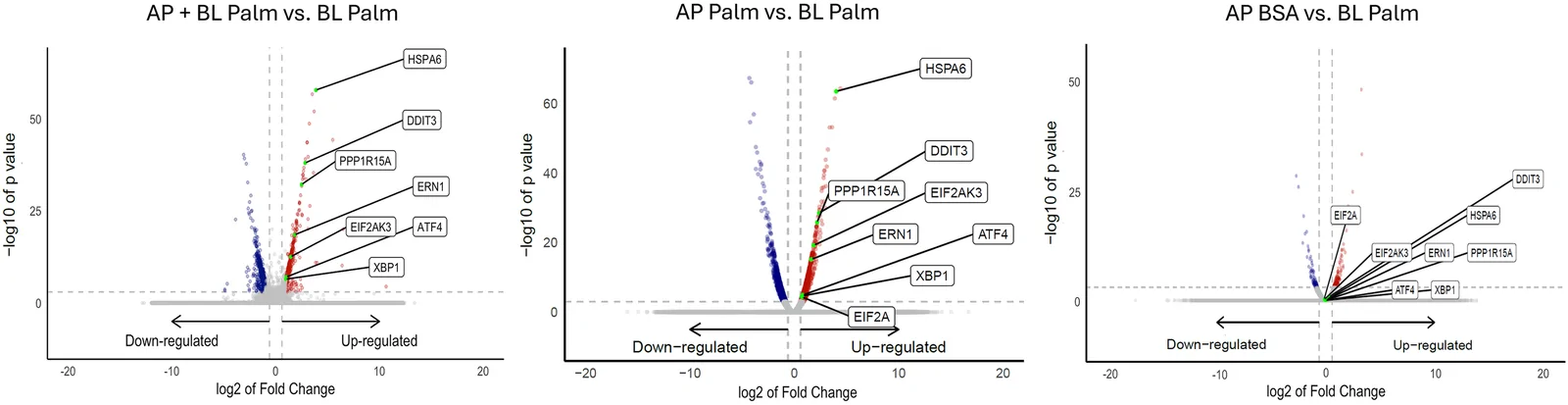
ER stress is induced by AP palmitate exposure. (**A**) Volcano plot for gene expression resulting from AP + BL versus BL palmitate (100 μM complexed with 0.2% BSA, 16 hours, 37°C), to simulate pathophysiologic conditions, with labeling of multiple upregulated ER stress genes. (**B**) Volcano plot for gene expression resulting from AP versus BL palmitate (100 μM complexed with 0.2% BSA, 16 hours, 37°C), to determine isolated effects of AP palmitate. (**C**) Volcano plot for gene expression resulting from AP BSA (0.2%, 16 hours, 37°C) versus BL palmitate (100 μM complexed with 0.2% BSA, 16 hours, 37°C). Gene names and corresponding proteins: ATF4 (activating transcription factor 4), DDIT3 (CCAAT/enhancer-binding protein homologous protein [CHOP]), EIF2A (eukaryotic translation initiation factor 2A), EIF2AK3 (protein kinase R–like endoplasmic reticulum kinase [PERK]), ERN1 (inositol-requiring enzyme 1 [IRE1]), HSPA6 (heat shock protein 70B), PPP1R15A (growth arrest and DNA damage–inducible protein 34 [GADD34]), XBP1 (X-box binding protein 1).

To screen for pathophysiologically relevant pathways, human proximal tubule cells were grown on permeable supports, which permitted isolated AP and/or BL additions (43). Because circulating palmitate is increased with diabetes (26), and albumin-bound palmitate is filtered in DKD, physiologic and pathologic conditions were modeled by comparison of incubation with BL versus AP + BL palmitate (non-covalently bound to albumin carrier). Bulk RNA-seq was conducted for the following conditions: (a) BL palmitate, (b) AP + BL palmitate, (c) AP palmitate, and (d) AP delipidated albumin control. Kyoto Encyclopedia of Genes and Genomes (KEGG) analysis from AP + BL versus BL palmitate experiments revealed that ER stress represented the most prominent pathway (Supplemental Figure 1; supplemental material available online with this article; https://doi.org/10.1172/jci.insight.199699DS1). Gene set enrichment analyses also revealed high scores for the unfolded protein response (Supplemental Figure 2). The volcano plots in Figure 2 highlight ER stress transcript expression. Importantly, the pattern was almost identical for AP + BL versus BL palmitate (Figure 2A) and AP versus BL palmitate (Figure 2B), indicating that ER stress is driven by AP palmitate uptake. No difference in ER stress transcript expression was noted between AP albumin carrier and BL palmitate groups (Figure 2C), further indicating that AP palmitate is the source of ER stress. Heatmaps show that AP + BL palmitate altered the expression of many ER stress transcripts, most of which were upregulated compared with BL palmitate (Supplemental Figure 3).

### ER stress with in vitro and in vivo models.

**Figure 3:**
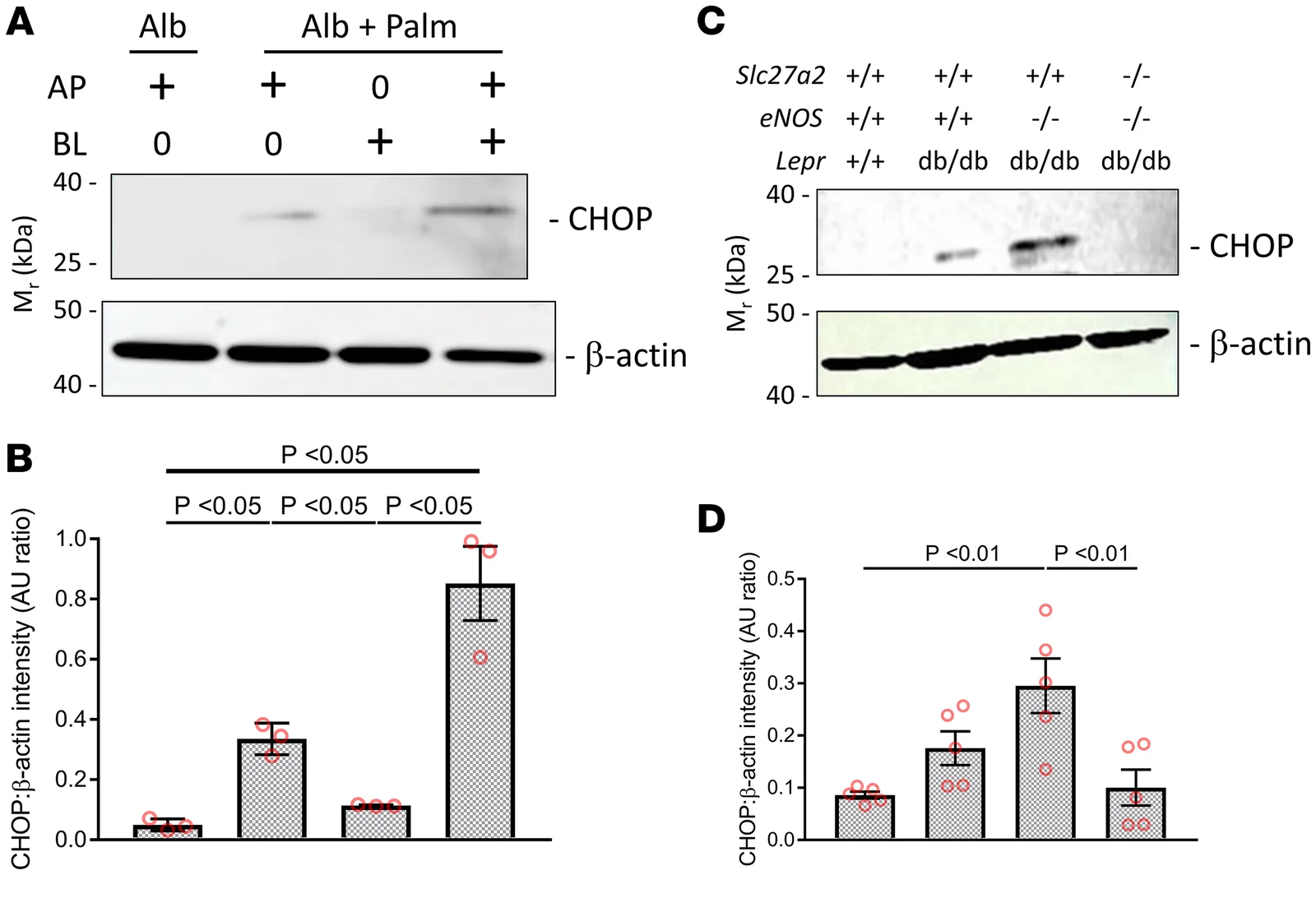
ER stress with in vitro and in vivo models. (**A**) AP or BL surfaces of human proximal tubule cells on permeable supports were incubated with albumin (0.2%) or albumin (0.2%) complexed with palmitate (100 μM) for 16 hours at 37°C. Lysates were probed for CHOP and β-actin expression by immunoblot analysis, as described in Methods (*N* = 3 biologically independent samples). (**C**) Kidney cortex lysates from mice with indicated genotypes were probed for CHOP and β-actin expression by immunoblot analysis, as described in Methods (*N* = 5 biologically independent samples). Data were quantitated from digitized images using ImageJ software (NIH), shown in histograms as mean ± SEM below representative blots, and analyzed by 1-way ANOVA and Tukey’s post hoc test for multiple comparisons (**B** and **D**). *Slc27a2*, FATP2 gene; *eNOS*, endothelial nitric oxide gene; *Lepr*, leptin receptor gene.

To test whether the RNA-seq data reflect protein expression, the in vitro palmitate experiments were repeated, and cell lysates were probed for expression of CHOP, which is a downstream effector of ER stress (44). Figure 3, A and B, shows that CHOP was most abundant with AP + BL palmitate but was also detected in the AP-palmitate-only condition. Long-chain FAs are taken up by FATP2, which is exclusively expressed on proximal tubule AP membranes in the kidney (21). AP + BL palmitate–induced CHOP expression was inhibited by the FATP2 inhibitor Lipofermata (MedChemExpress) (45) (Supplemental Figure 4). In vivo, CHOP was abundantly expressed in renal cortex lysates from *db/db eNOS^–/–^* mice, which phenocopy DKD, but not in lysates from *db/db eNOS^–/–^* mouse kidneys with FATP2 gene deletion (Figure 3, C and D). Taken together, the data support that FA uptake by AP FATP2 stimulates ER stress.

### AP palmitate causes ER fragmentation.

**Figure 4:**
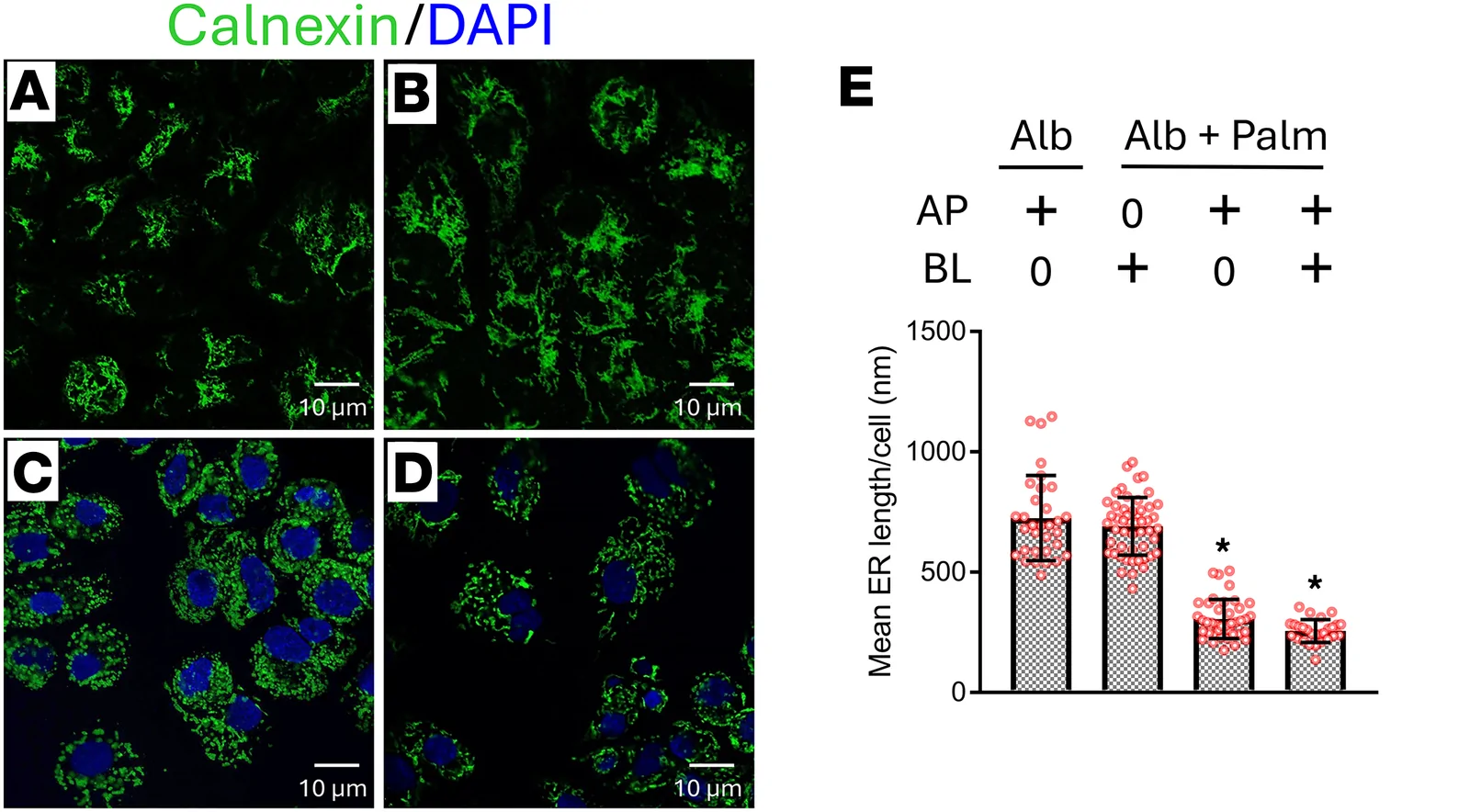
AP palmitate causes ER fragmentation. (**A**–**D**) ERs were labeled with calnexin antibodies and nuclei counterstained with DAPI from proximal tubule cells on permeable supports exposed to 0.2% AP albumin carrier (**A**), BL palmitate (100 μM, 16 hours, 37°C) complexed with 0.2% albumin (**B**), AP palmitate (100 μM, 16 hours, 37°C) complexed with 0.2% albumin (**C**), or AP + BL palmitate (100 μM, 16 hours, 37°C) complexed with 0.2% albumin (**D**). Scale bars: 10 μm. (**E**) AP or BL surfaces of human proximal tubule cells on permeable supports were incubated with albumin (0.2%) or albumin (0.2%) complexed with palmitate (100 μM) for 16 hours at 37°C. Cells were fixed and labeled for ER with calnexin antibodies as in **A**–**D**, and ER length was quantified, as described in Methods. Each symbol represents the mean of multiple ER measurements from a single cell and 3 experiments. Data are mean ± SD. *P < 0.001 compared with BL Alb + Palm group by ANOVA and Tukey’s post hoc test for multiple comparisons.

ER stress may be associated with altered ER morphology, including a widened lumen to accommodate chaperone-mediated unfolded protein response, and shortening by reticulophagy to delete dysfunctional ER (30, 46–48). Changes in ER morphology, in response to palmitate, were assessed by measurement of length of tubular ER, the site of lipid synthesis (49). ERs from AP or AP + BL palmitate–treated cells were markedly shorter (Figure 4, A–D), with a mean length of about 200 nm compared with about 700 nm in albumin carrier– and BL palmitate–treated controls (Figure 4E), which is consistent with other reports (50).

### In vivo and in vitro models are associated with decreased LD biogenesis.

**Figure 5:**
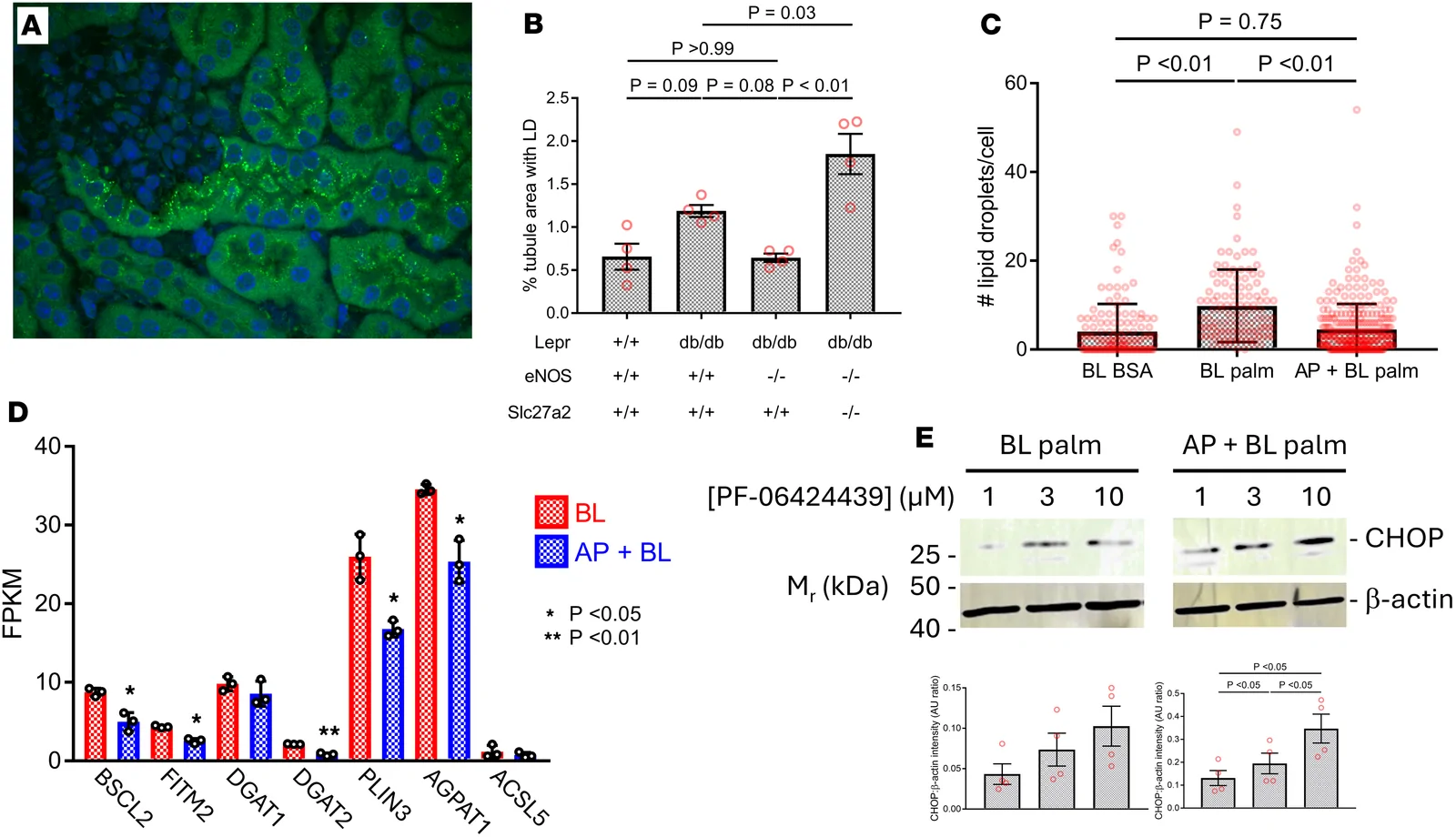
In vivo and in vitro models are associated with decreased LD biogenesis. (**A**) Representative kidney cortex section from db/db mouse stained for LDs with LipidSpot, as described in Methods. Original magnification, ×400. (**B**) Quantification by ImageJ of LDs from kidney cortex of mice (4 non-consecutive sections from 4 mice per genotype). Data are percentage tissue area that contained LDs from an individual mouse and are expressed as mean ± SEM, and were analyzed by 1-way ANOVA and Tukey’s post hoc test for multiple comparisons. (**C**) BL and/or AP surfaces of human proximal tubule cells on permeable supports were incubated with albumin (0.2%) or albumin (0.2%) complexed with palmitate (100 μM) for 16 hours at 37°C. Cells were fixed, labeled for LDs with LipidSpot as described in Methods, and quantified using ImageJ. Each symbol represents mean LD number in 110 to 289 individual cells per group from 3 separate experiments. Data are expressed as mean ± SD. Data from multiple groups were analyzed by 1-way ANOVA and Tukey’s post hoc test for multiple comparisons. (**D**) LD biogenesis gene transcript expression in human proximal tubule cells treated with AP + BL versus BL palmitate (100 μM complexed with 0.2% BSA, 16 hours, 37°C). Data are mean ± SD from 3 experiments. *P* values reflect AP + BL versus BL palmitate comparisons by 2-tailed *t* test. FPKM, fragments per kilobase transcript per million mapped reads; BSCL2, seipin; FITM2, fat storage–inducing transmembrane protein-2; DGAT, diacylglycerol acyltransferase; PLIN3, perilipin-3; AGPAT1, 1-acyl-*sn*-glycerol-3-phosphate acyltransferase-α; ACSL5, long-chain acyl-CoA synthetase-5. (**E**) Human proximal tubule cells were pretreated for 1 hour with the DGAT2 inhibitor PF-06424439 at indicated concentrations, then treated with AP + BL or BL palmitate (100 μM complexed with 0.2% BSA, 16 hours, 37°C), and probed for CHOP or β-actin by immunoblot analysis, as described in Methods (*N* = 3 biologically independent samples). Immunoblot data were quantitated using ImageJ software and are shown in histograms below representative blots. Data are depicted as mean ± SEM and were analyzed by 1-way ANOVA and Tukey’s post hoc test for multiple comparisons.

The next series of experiments explored the fate of FAs in the context of ER stress initiation. Because palmitate is not well incorporated into triacylglycerol (27–29), and excess intracellular FAs can induce ER stress (29, 51), we tested whether bilateral palmitate affects LD biogenesis. Figure 5A demonstrates LDs in *db/db* mouse kidney, which accumulate predominantly on the AP side of the proximal tubule. LD density was decreased in proximal tubules from *db/db eNOS^–/–^* mice, which phenocopy DKD, and increased in *db/db eNOS^–/–^ Slc27a2^–/–^* kidneys (Figure 5B and Supplemental Figure 5). These data are in agreement with conditional *Slc27a2^–/–^* mice and inducible DKD (Figure 1H) and support that sequestration of FAs and FA metabolites in LDs protects proximal tubules against cytotoxicity. In vitro, cells exposed to AP + BL palmitate contained fewer LDs compared with cells treated with BL palmitate only (Figure 5C). These data are consistent with decreased expression of transcripts encoding LD biogenesis proteins in the AP + BL palmitate–treated group (Figure 5D). The in vivo and in vitro results indicate that AP FA uptake leads to decreased LD number. Figure 5E demonstrates that blocking LD biogenesis with the diacylglycerol acyltransferase-2 inhibitor PF-06424439, at concentrations that exceed the IC_50_ = 14 nM (52), but less than the 30 to 50 μM doses used in some studies (29), enhanced ER stress in response to AP + BL palmitate, suggesting that diminished sequestration of FAs and lipotoxic intermediates by LDs precipitates ER stress.

### Mouse renal cortex ceramide concentrations.

**Figure 6:**
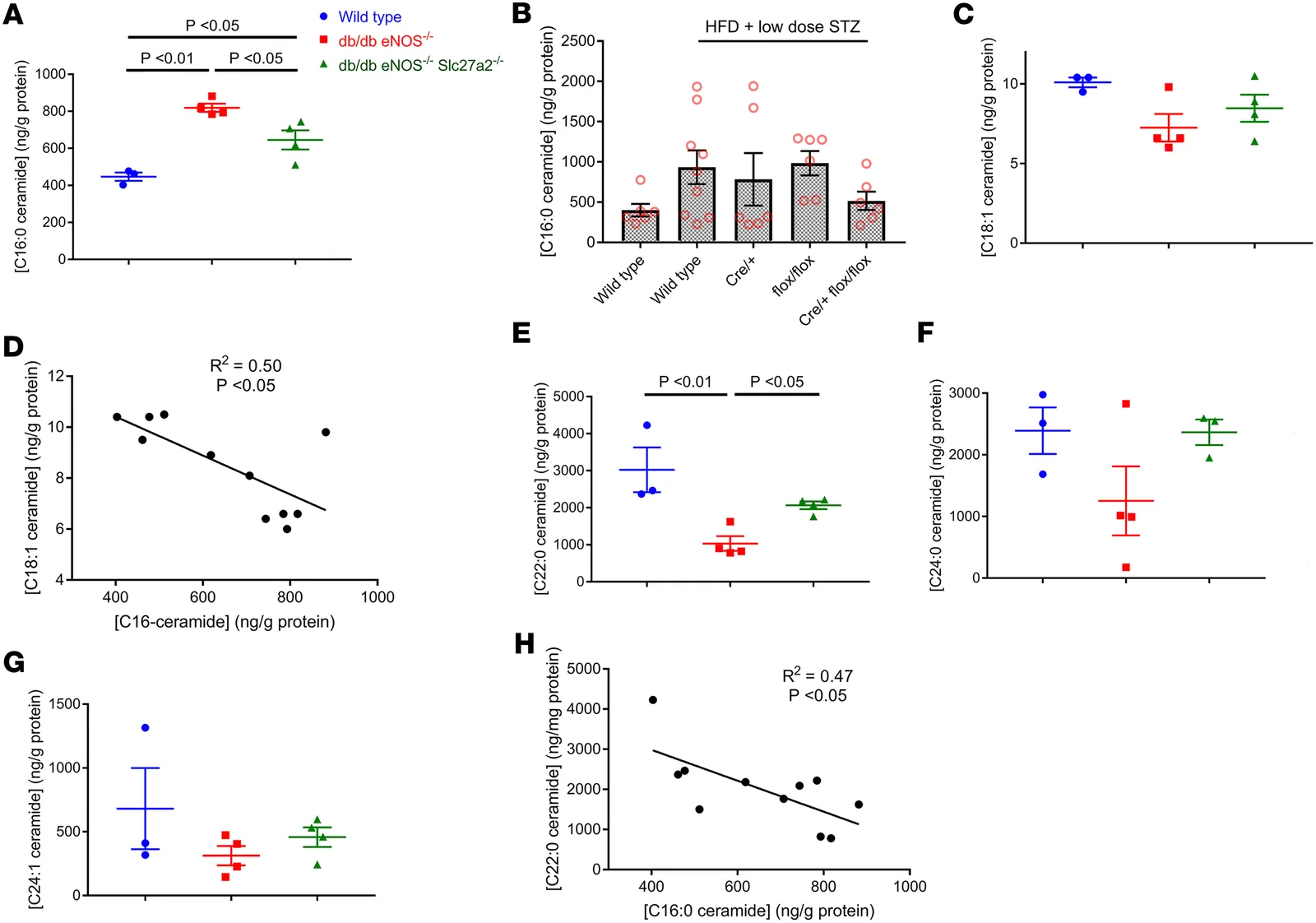
Mouse renal cortex ceramide concentrations. Kidney cortex from mice with the indicated genotypes and exposures was snap-frozen, assayed for ceramides by LC-MS/MS as described in Methods, and normalized to protein content. (**A** and **B**) C16:0 ceramide. (**C**) C18:1 ceramide. (**D**) Plot illustrating relationship between long-chain ceramides with saturated (C16:0) and monounsaturated acyl chains. (**E**) C22:0 ceramide. (**F**) C24:0 ceramide. (**G**) C24:1 ceramide. (**H**) Plot illustrating relationship between ceramides with very long (C22:0) and long (C16:0) acyl chains. Data for **A**–**C** and **E**–**G** are depicted as mean ± SEM and were analyzed by 1-way ANOVA and Tukey’s post hoc test for multiple comparisons. Data from **D** and **H** were analyzed by linear regression, and *P* values indicate that slopes were significantly different. Note different *y* axis ranges with each ceramide species.

Because ceramides are synthesized in the ER and have been implicated in ER stress and DKD (38), we queried whether palmitate exposure leads to altered intracellular ceramide concentrations. C16:0 ceramide was increased in *db/db eNOS^–/–^* kidneys and decreased in kidneys from *db/db eNOS^–/–^ Slc27a2^–/–^* mice (Figure 6A). There was a decreased C16:0 ceramide trend in conditional proximal tubule *Slc27a2^–/–^* mice with inducible DKD (Figure 6B). Interestingly, there was an inverse correlation between C16:0 and unsaturated C18:1 ceramide (Figure 6D), as well as very-long-chain C22:0 ceramide (Figure 6, E and H), which are cytoprotective (53–55). C18:1, C24:0, and C24:1 (Figure 6, C, F, and G) ceramide concentrations are also shown.

### Ceramide concentrations in proximal tubule cells.

**Figure 7:**
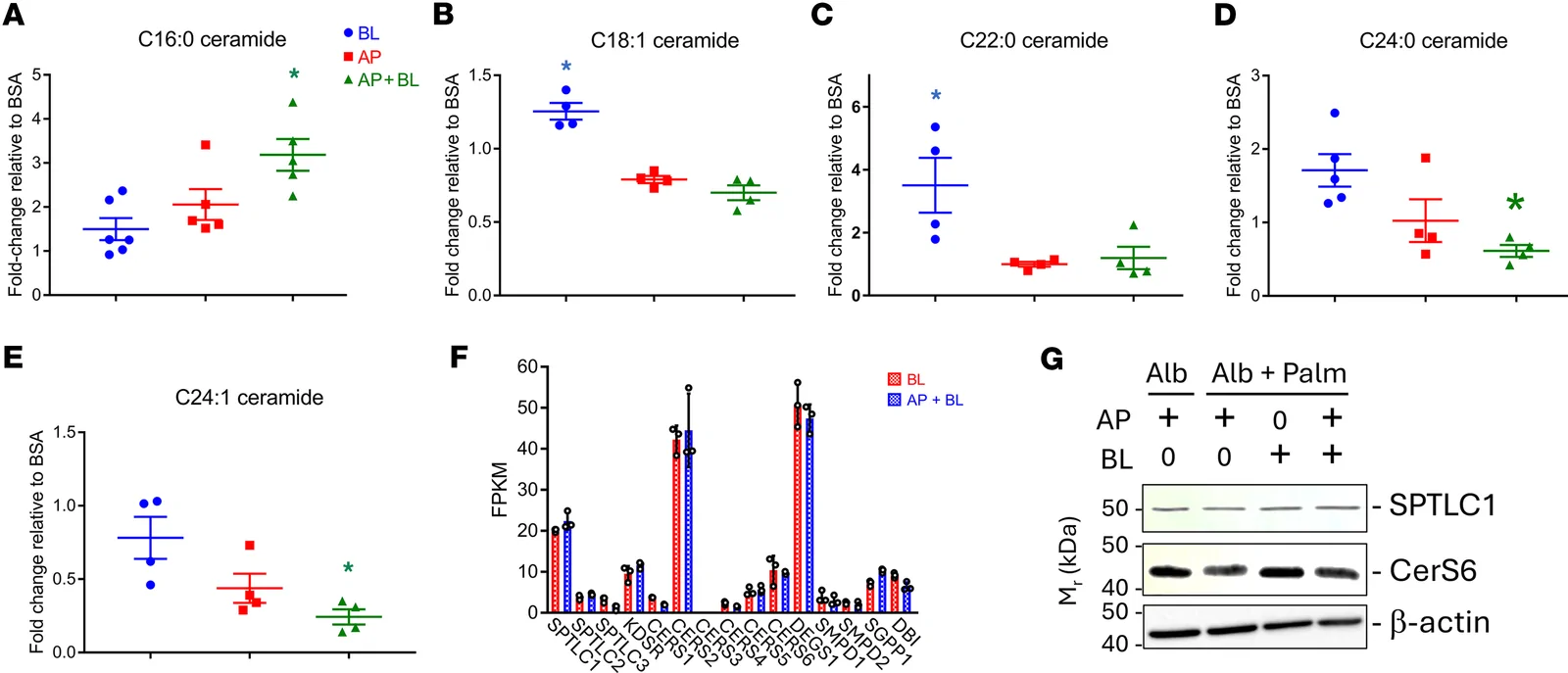
Ceramide concentrations in proximal tubule cells. (**A**–**E**) AP and/or BL surfaces of human proximal tubule cells on permeable supports were incubated with palmitate (100 μM complexed with 0.2% BSA, 16 hours, 37°C). Cells were scraped from permeable supports into glass vials, frozen, and assayed for ceramides by LC-MS/MS as described in Methods. Data are expressed as ceramide concentrations normalized to values obtained in cells exposed to albumin (BSA) carrier only. Data for **A**–**E** are depicted as mean ± SEM and were analyzed by 1-way ANOVA and Tukey’s post hoc test for multiple comparisons. Blue asterisk, *P* <0.01 between BL and AP + BL groups by ANOVA. Green asterisk, *P* <0.01 between AP + BL and other groups by ANOVA. (**F**) Ceramide synthesis gene transcript expression in human proximal tubule cells treated with AP + BL versus BL palmitate (100 μM complexed with 0.2% BSA, 16 hours, 37°C). Data are mean ± SD from 3 experiments. All *P* values > 0.05 by 2-tailed *t* test. FPKM, fragments per kilobase transcript per million mapped reads; SPTLC1, serine palmitoyltransferase long-chain base subunit 1; KDSR, 3-ketodihydrosphingosine reductase; CERS, ceramide synthase; DEGS1, delta 4-desaturase sphingolipid-1; SMPD1, sphingomyelin phosphodiesterase-1; SMPD2, sphingomyelin phosphodiesterase-2; SGPP1, sphingosine-1-phosphate phosphatase-1; DBI, diazepam-binding inhibitor, also known as acyl-CoA–binding protein. (**G**) Human proximal tubule cells were treated with AP + BL or BL palmitate (100 μM complexed with 0.2% BSA, 16 hours, 37°C) and probed for expression of indicated proteins by immunoblot analysis, as described in Methods (*N* = 3 biologically independent samples).

In vitro, C16:0 ceramide was slightly increased with AP palmitate incubation and more markedly increased with AP + BL palmitate (Figure 7A). As in the in vivo DKD models, reciprocal decreases in C18:1 ceramide and very-long-chain ceramides were also observed under the AP + BL palmitate conditions (Figure 7, B–E). However, the increased C16:0 ceramide concentrations were not accompanied by changes in mRNA expression of ceramide synthesis enzymes (Figure 7F).

Serine palmitoyltransferase (SPT) catalyzes the rate-limiting step in ceramide synthesis by combining palmitoyl-CoA with l-serine to form sphinganine. In a subsequent step, ceramide synthase 5 and ceramide synthase 6 (CerS6) catalyze the formation of C16:0 ceramide synthesis by transfer of palmitoyl-CoA to the sphingoid base. No difference in SPTLC1 subunit or CerS6 protein expression was observed between any of the palmitate incubation conditions (Figure 7G). Figures 6 and 7 indicate that AP palmitate uptake leads to increased C16:0 ceramide accumulation, as a result of enhanced C16:0 FA substrate availability rather than ceramide synthesis enzyme induction. Sphingomyelinases may also contribute to ceramide accumulation, but mRNA expression of acid and neutral sphingomyelinases (SMPD1 and SMPD2), the major sphingomyelinases for C16:0 ceramide generation, was not different in AP + BL versus BL palmitate or BSA carrier conditions (Figure 7F).

### Ceramides are a cause and not an effect of ER stress.

**Figure 8:**
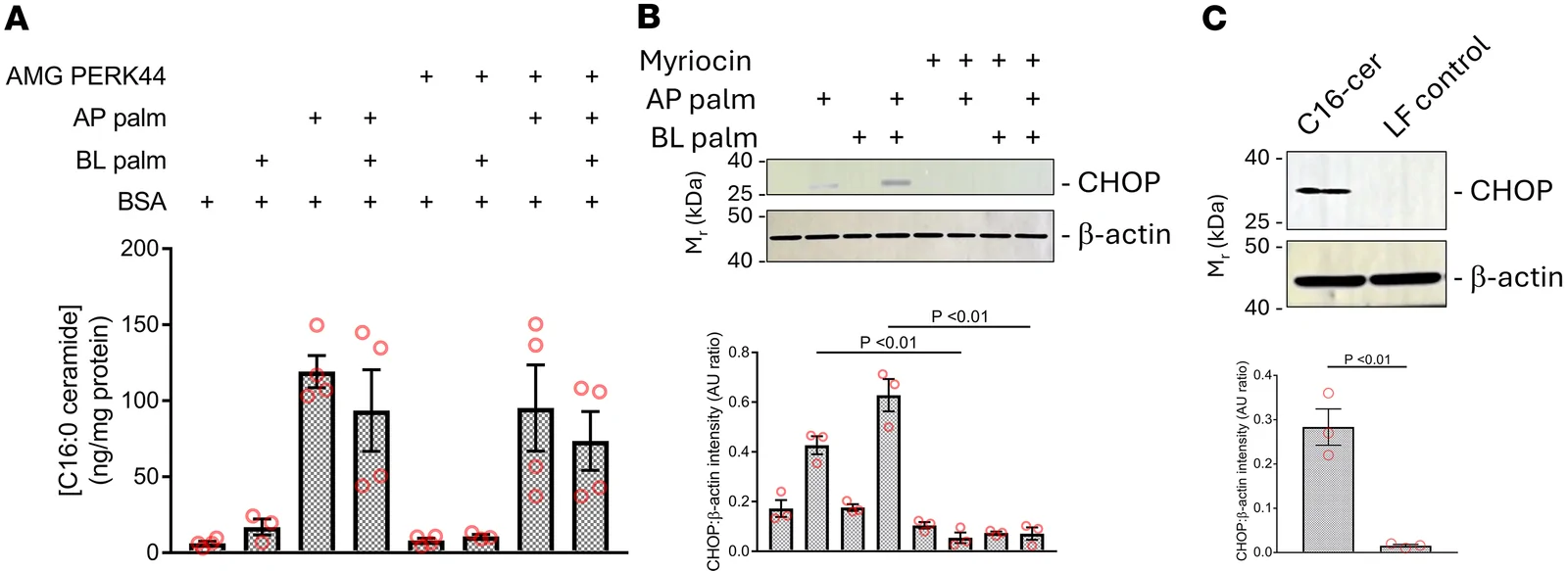
Ceramides are a cause and not an effect of ER stress. (**A**) Human proximal tubule cells were pretreated with the PERK inhibitor AMG PERK44 (1 μM, 1 hour, 37°C), then treated with AP and/or BL palmitate (100 μM complexed with 0.2% BSA, 16 hours, 37°C). Cells were scraped from permeable supports into glass vials, frozen, and assayed for ceramides by LC-MS/MS as described in Methods. Results are mean ± SEM from 4 experiments. (**B**) Human proximal tubule cells were pretreated with myriocin (10 μM, 1 hour, 37°C), then with AP and/or BL palmitate (100 μM complexed with 0.2% BSA, 16 hours, 37°C). Cell lysates were probed for CHOP or β-actin by immunoblot analysis, as described in Methods (*N* = 4 biologically independent samples). (**C**) Human proximal tubule cells on permeable supports were incubated with Lipofectamine 3000 (LF) with or without C16:0 ceramide (2–4 hours, 37°C in humidified 5% CO_2_ incubator). Cell lysates were probed for CHOP or β-actin by immunoblot analysis, as described in Methods (*N* = 3 biologically independent samples). Immunoblot data were quantitated using ImageJ software and are shown in histograms below representative blots. Data are depicted as mean ± SEM and were analyzed by unpaired 2-tailed *t* test.

Ceramides are synthesized in the ER (56) and can be the cause or the effect of ER stress (34, 57–59). ER stress is mediated by 3 pathways, which are initiated by protein kinase R–like ER kinase (PERK), activating transcription factor 6 (ATF6), or inositol-requiring enzyme 1 (IRE1). Of these, the PERK pathway was the most highly upregulated by AP palmitate incubation (Figure 2 and Supplemental Figure 3). To test the effect of ER stress on ceramide concentration, cells were preincubated with the PERK inhibitor AMG PERK44. Supplemental Figure 6 shows that AMG PERK44 inhibited CHOP formation in AP or AP + BL palmitate–incubated cells. Figure 8A shows that AMG PERK44 had no effect on intracellular C16:0 ceramide concentrations, suggesting that ER stress was not the cause of increased ceramides.

### C16:0 ceramide causes ER membrane phase transition to a more ordered state.

To test whether ceramide incorporation into ER membranes caused ER stress, ceramide synthesis was blocked with the SPT inhibitor myriocin. Supplemental Figure 7A shows dose-dependent myriocin inhibition of C16:0 ceramide formation. Myriocin modestly enhanced LD formation in cells exposed to BL or AP + BL palmitate (Supplemental Figure 7B), suggesting either that the effect was due to incomplete SPT inhibition (Supplemental Figure 7A) or that defective lipid storage only partially explains ceramide toxicity. Figure 8B shows once again that AP + BL > AP >> BL palmitate stimulates ER stress. This effect was inhibited by myriocin, implying that ceramides are a cause rather than an effect of ER stress.

**Figure 9:**
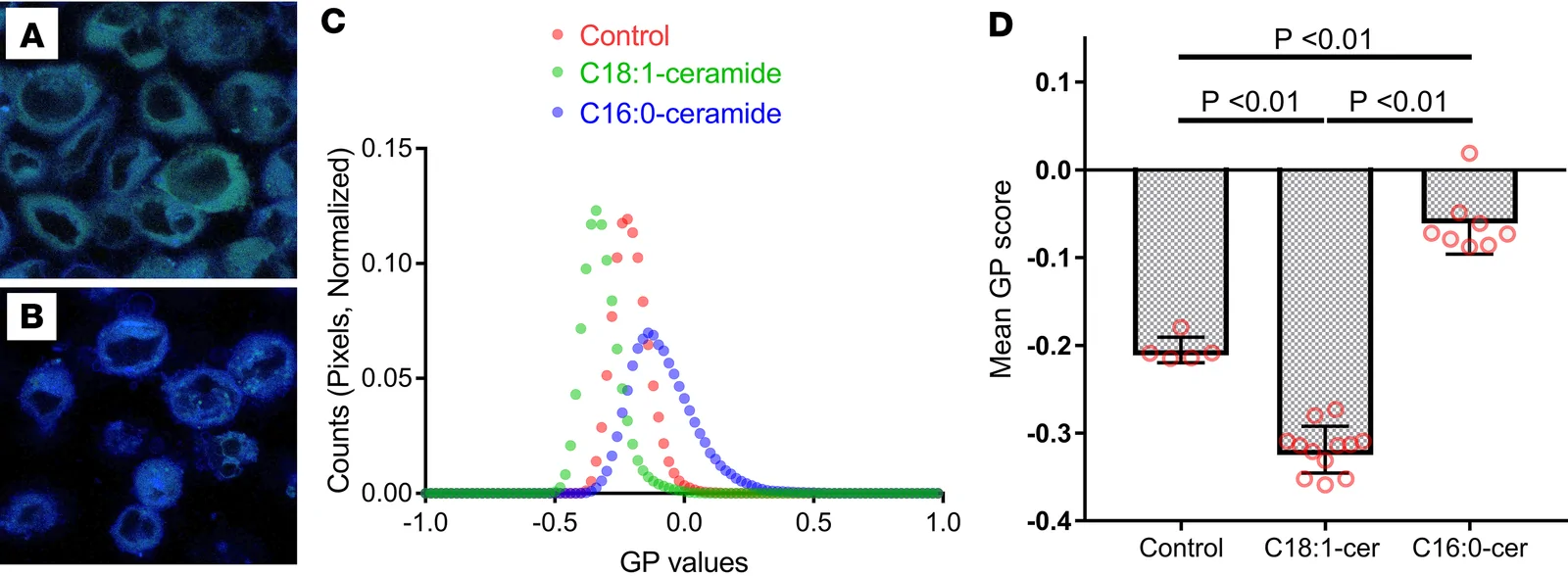
C16:0 ceramide causes ER membrane phase transition to a more ordered state. Human proximal tubule cells on permeable supports were treated with 0.2% albumin carrier (**A**) or bilateral 10 μM C16:0 ceramide complexed with 0.2% albumin (**B**) for 16 hours at 37°C. Images show C-laurdan fluorescence of merged green (λ = 490 nm)/blue (λ = 440 nm) wavelength. Original magnification, ×400.(**C**) To assess ER membrane fluidity, cells on permeable supports were transfected with either C18:1 or C16:0 ceramides, and then colabeled with C-laurdan and ER tracker. C-laurdan fluorescence in colocalized ER was determined by ImageJ (values from 5–13 experiments per condition). Negative generalized polarization (GP) values correspond to the more liquid (disordered) phase, whereas less negative or positive GP values correspond to the more gel-like (ordered) phase. (**D**) Mean peak GP score ± SEM is shown; data were analyzed by 1-way ANOVA and Tukey’s post hoc test for multiple comparisons.

The direct effect of ceramides on ER stress was then tested by Lipofectamine-assisted transfection, which was verified with NBD-labeled C12 ceramide (Supplemental Figure 8, A–D) and liquid chromatography-tandem mass spectrometry (LC-MS/MS) quantification (Supplemental Figure 8E). The increase in Lipofectamine-transduced C16:0 ceramide was associated with ER stress (Figure 8C). Ceramides have been implicated as inducers of ER stress by decreasing ER membrane fluidity, and induced proximity-mediated oligomerization of PERK and IRE1 (39–41). To test for this possibility, AP + BL C16:0 ceramide–loaded cells were stained with C-laurdan, which enables assessment of organellar membrane phase transition (60, 61). Figure 9A demonstrates predominant green C-laurdan fluorescence at baseline, representing relatively fluid organellar membrane domains, whereas introduction of AP + BL C16:0 ceramide caused C-laurdan fluorescence emission to shift from green to blue spectra (Figure 9B), signifying more ordered organellar membranes. Quantitative ER-restricted C-laurdan fluorescence revealed phase transition to relatively more ordered membranes with C16:0 compared with C18:1 ceramide loading (Figure 9, C and D), which reflects decreased ER membrane fluidity from C16:0 ceramide packing.

## Discussion

DKD is characterized by both glomerular and tubulointerstitial pathology. Although tubular atrophy is a superior predictor of DKD progression (4–7), the tubular pathophysiology is less well studied. The most widely accepted view is that glomerular filtration barrier dysfunction permits excess albumin, with non-covalently bound long-chain FAs, to be aberrantly filtered. Albumin and FAs dissociate within the lumen, albumin is reabsorbed by the megalin/cubilin complex, and the FAs are taken up by AP proximal tubule transport (62). In this report we show that proximal tubule–specific deletion of FATP2, which is exclusively expressed in the AP proximal tubule membrane in kidney (21), restored GFR in mice that phenocopy DKD, thereby illustrating the primacy of FATP2 as a mediator of lipotoxicity in the pathogenesis of DKD. In vivo and in vitro studies to define downstream mechanisms of lipotoxicity demonstrated that the excess intracellular FAs (palmitate) were not sequestered by LDs, but instead were incorporated into long-chain ceramides, which were inserted into ER membranes, leading to increased ER membrane order and lipid bilayer stress.

Proximal tubule ER stress has previously been described, both in vivo and in cultured proximal tubule cells under simulated diabetic conditions (63, 64). In contrast with reports in cultured cells (29), but consistent with human kidney biopsy data (65), we noted that PERK and IRE1, but not ATF6, pathways of ER stress were stimulated. We speculate that adherence with the human data may be due to the maintenance of proximal tubule cells on permeable supports, which preserved differentiation, and thereby more accurately mimicked in vivo conditions. Studies in cells on permeable supports also permitted analysis of differential AP versus BL effects, which can only be surmised using in vivo models, or in vitro with cells on plastic surfaces. BL palmitate incubation, to simulate constitutive baseline FA oxidation as the main source of ATP generation, did not stimulate ER stress–associated genes, whereas AP + BL palmitate exposure, to mimic pathologic conditions characterized by constitutive BL + AP FA uptake following glomerular filtration of albumin-bound FAs, caused robust ER stress.

The finding that ER stress gene stimulation was virtually identical with AP or AP + BL palmitate implicates AP membrane FA uptake–regulated ER stress in the pathogenesis of tubular atrophy. Of known FA transporters, FATP2 and KIM-1 are the most relevant (21, 22, 66), since both proteins localize exclusively to the AP proximal tubule membrane. While KIM-1 was not evaluated here, global FATP2 deletion ameliorated ER stress in *db/db eNOS^–/–^* mice (Figure 3, C and D), and conditional proximal tubule FATP2 deletion in an HFD plus low-dose STZ model reversed GFR declines (Figure 1), suggesting that AP FATP2 directs lipotoxicity.

FATP2 is a long- and very-long-chain FA transporter. The long-chain FA palmitate is increased in plasma from patients with diabetes (26), and palmitate can be metabolized to multiple lipid intermediates. We focused on ceramides because of their potential importance in DKD pathogenesis, and because palmitoyl-CoA reaction with l-serine in ER, to form sphinganine, is the rate-limiting step in ceramide formation. C16:0 ceramide was increased, both in tissue from mice that phenocopy DKD and in proximal tubule cells exposed to AP + BL palmitate. Based on data derived from inhibitors of ceramide synthesis, ER stress, and LD biogenesis, we conclude that palmitate is not well incorporated into LDs (27), and the resulting palmitate-derived ceramides are a cause rather than an effect of ER stress. Interestingly, sphingolipids have been associated with ATF6-mediated ER stress (67), which was not increased in our model. Rather, we noted ceramide increases in the context of PERK-mediated, and to a lesser extent IRE1-mediated, ER stress.

In experiments to mimic FA-induced increases in intracellular ceramides, we observed baseline ER C-laurdan generalized polarization (GP) values that were comparable to prior reports and reflect more loosely packed, less rigid membranes compared with other organelles (68, 69). C16:0 and C18.1 ceramides decreased and increased ER membrane fluidity, respectively. Other membrane lipids undoubtedly also contribute to ER lipid bilayer stress, and their identification will require further investigation. Extracellular ceramides have been shown to induce ER stress in vitro (58, 70), with subsequent increased ceramide-ER colocalization and decreased fluidity of inner and plasma membranes (70). However, quantitative ER membrane C-laurdan fluorescence was not determined. Furthermore, because secreted ceramides were packaged in extracellular vesicles (58) or bound to lipoproteins (70), the applicability to DKD pathogenesis is questionable. While smaller lipoproteins (diameter range = 10–80 nm) could conceivably cross the damaged glomerular filtration barrier and become exposed to AP membranes of recipient proximal tubule cells, sieving studies in humans and animal models of DKD demonstrate that the filtration fraction is still very low for dextrans of greater than 10 nm diameter (71, 72). Exosomes (30–150 nm diameter) and microvesicles (100–1,000 nm diameter) are even larger than lipoproteins, and therefore unlikely to undergo glomerular filtration.

C16:0 ceramide has a conical shape, which promotes membrane rigidity and gel phase transition (73, 74). Reduced ER membrane fluidity has been associated with oligomerization and activation of PERK and IRE1 by induced proximity lipid bilayer stress, which is independent of unfolded proteins within the ER lumen (39–41). The proposed mechanism is that increased saturated membrane lipids weaken the interactions between transmembrane protein domains. The preferential exclusion of transmembrane proteins from the gel phase then increases local density in the diminished liquid phase, favoring dimerization (40, 75).

The increased C16:0 ceramide content in both *db/db eNOS^–/–^* mouse kidneys and cells exposed to bilateral palmitate was accompanied by a decrease in very-long-chain ceramides. This reciprocal pattern of long-chain/very-long-chain ceramides has been described in other tissues (53, 54, 76). Potential biochemical mechanisms for the inverse relationship are compensatory changes in ceramide synthase or FA elongase expression. We noted no changes in ceramide synthase isoform transcript expression, but we did observe decreased ELOVL2 and ELOVL6 expression (Supplemental Figure 9), consistent with decreased very-long-chain ceramide synthesis. In general, long-chain ceramides are associated with cytotoxicity, whereas very-long-chain ceramides promote cell survival (53–55). The proposed biophysical mechanism is related to amphipathic helical domains within PERK and IRE1 interacting with compressed, less fluid membrane lipid bilayers (41). Long-chain ceramides are more amenable to compression compared with very-long-chain ceramides, although carbon bond saturation is also a factor, with greater flexibility of unsaturated ceramides.

An important technical finding is the establishment of a method for testing the direct effect of ceramides on intracellular organelle function. Previous methods relied on incubation of ceramides in dodecane/ethanol buffer (58), which is effective primarily for introduction of shorter-chain ceramides. We confirmed that these methods achieved minimal increases in intracellular C16:0–24:0 ceramides. However, by adapting a Lipofectamine-assisted transfection protocol, which is typically used for introducing cDNAs into cells, we achieved intracellular concentrations of C16:0 ceramide, which were comparable to pathologic levels observed with palmitate incubation.

The conditions associated with ER stress (AP or AP + BL palmitate) also resulted in substantially shorter ER length. Palmitate has been associated with increased ER volume, through membrane lipid biogenesis (30, 46, 47, 77), to accommodate unfolded proteins and chaperones as part of the ER stress response (48, 67). However, palmitoyl-CoA and membrane phospholipids with saturated acyl chains also facilitate ER fission by enhancing membrane rigidity (78, 79), as was observed in our studies (Figure 9). In healthy cells, ER stress may be accompanied by ER fragmentation through ER-phagy, which facilitates protein degradation and restoration of dilated ER to the normal size (48). The dynamic control of ER length is mediated by the balance between fission, which is regulated by reticulons and reticulon-like proteins, and atlastin-mediated fusion (50, 80). However, no differences in expression of genes encoding these proteins were observed in our RNA-seq dataset (not shown), implying that sustained palmitate exposure, to model disease, stimulated ER fragmentation by a mechanism other than ER-phagy. Fragmented ERs were observed in cells that did not exhibit chromatin degradation or plasma membrane blebbing (Figure 4) or altered expression of transcripts that encode apoptosis proteins (caspases-3/9, BAX/BAK/BIM/BIK, PUMA; not shown), suggesting that the cause was also not apoptosis. Further studies will be required to clarify the mechanism.

FATP2 functions as a salvage FA transporter under normal circumstances but is a major mediator of proximal tubule lipotoxicity under pathologic conditions (22). The fate of reabsorbed FAs, and the intracellular mechanisms of lipotoxicity, had not previously been explored. In vivo and in vitro experimental data indicate that AP (but not BL) proximal tubule FA uptake, which exceeds the capacity for LD incorporation, caused ceramide-induced ER membrane phase transition to a more gel-like state, and subsequent cytotoxicity from ER stress.

## Methods

### Sex as a biological variable.

With the exception of high-fat diet experiments, in which only male mice develop obesity, no differences were noted between male and female mice for any parameters. Therefore, equal numbers of male and female mice were used in all other experiments.

### Mice.

Conditional deletion of the FATP2 gene (*Slc27a2*) from proximal tubules was achieved by crossing of female *Ggt1*-Cre mice (The Jackson Laboratory) (81) with male floxed *Slc27a2* mice (gift from Dmitry Gabrilovich, AstraZeneca, Gaithersburg, Maryland, USA) (22, 81) on congenic C57BLKS/J genetic backgrounds, to generate Cre^+/–^ *Slc27a2^fl/fl^* as well as genotype control Cre*^+/–^* *Slc27a2^+/+^* and *Slc27a2^fl/fl^* mice. Efficiency of *Slc27a2* deletion has been previously described. To simulate kidney disease from inducible type 2 diabetes, 6-week-old male mice were fed an HFD (Teklad TD.06414, 60% calories from fat; Harlan Laboratories) for 6 months. At the 3-month time point, mice were injected with low-dose streptozotocin (40 mg/kg i.p. on 2 consecutive days), as previously described (22). The genetic models of kidney disease from type 2 diabetes (*Lepr^db/db^* mice with global *eNOS* gene deletion; *db/db eNOS^–/–^*) (22, 83) and *db/db eNOS^–/–^* mice with global FATP2 gene deletion (21, 84) (*db/db eNOS^–/–^ Slc27a2^–/–^*) have been previously characterized and described (22). Attempts to create *db/db eNOS^–/–^* mice with conditional proximal tubule FATP2 gene deletion (*Ggt1*-Cre^+/–^ *Slc27a2^fl/fl^ db/db eNOS^–/–^*) were consistently confounded by germline Cre contamination following intercrosses between *Ggt1*-Cre^+/–^ *Slc27a2^fl/+^ Lepr^db/+^ eNOS^+/–^* and *Slc27a2^fl/fl^ Lepr^db/+^ eNOS^+/–^* mice. Germline contamination was transmitted by both male and female *Ggt1*-Cre^+/–^ *Slc27a2^fl/+^ Lepr^db/+^ eNOS^+/–^* mice, consistent with a recent report regarding *Ggt1*-Cre *Fam134b^fl/fl^* mice (85).

### GFR measurements.

GFR was measured in mice by monitoring of fluorescently labeled sinistrin decay, as previously described (22), with minor modifications. Rather than subjecting mice to serial blood sampling, we used a TGFRII transdermal detection device and software (MediBeacon) for continuous fluorescence readings.

### Blood glucose measurements.

Blood glucose was assayed after fasting from 6:00 to 10:00 am. Tail vein blood glucose was assayed by glucometer, as previously described (42).

### Albuminuria measurements.

Spot urine samples were obtained from conscious mice in afternoons and at the time of sacrifice. Urine albumin was assayed by ELISA according to the manufacturer instructions (Albuwell M, Exocell) as previously described (22). Urine creatinine concentration was measured using a picric acid method according to manufacturer instructions (Creatinine Companion, Exocell).

### Cell culture.

Immortalized human proximal tubule cells were a gift from Ullrich Hopfer (Case Western Reserve University) (86). Each clonal cell line was maintained in keratinocyte serum-free medium (Life Technologies/GIBCO BRL) with 1% FBS and frozen at –80°C. Thawed cells were then grown on Transwell permeable supports (Corning) and passaged every 7–10 days after reaching 90% confluence.

### RNA-seq.

Cells were exposed to AP, BL, or AP plus BL 0.2% BSA complexed with palmitate (100 μM) or AP 0.2% BSA carrier for 16 hours at 37°C. Total RNA was extracted with TRIzol Reagent (Invitrogen) and quantified by NanoDrop (Thermo Fisher Scientific), and frozen aliquots were shipped to LC Sciences. RNA was assessed for sample quality, reverse-transcribed to cDNA, and sequenced on an Illumina NovaSeq 6000 machine, and data were analyzed using DESeq2 in the R package. Quantifications by fragments per kilobase of transcript per million mapped reads (FPKM) for more than 31,000 genes in 3 experiments per group were obtained. Gene set enrichment analyses (LC Sciences) included calculation of enrichment scores and false discovery rate *q* values after multiple-testing correction.

### Immunoblotting.

Immunoblotting methods have been previously described (87). Briefly, cells were lysed and proteins denatured in boiling buffer (125 mM Tris [pH 6.8], 2% SDS, 5% glycerol, 1% β-mercaptoethanol, 0.003% bromphenol blue) for 5 minutes. Samples (20 μg protein per lane) were resolved by SDS-PAGE and transferred to polyvinylidene difluoride membranes. Blots were blocked in 5% dried milk and probed with rabbit anti–human CHOP (ABclonal, A0221; 1:1,000 in 5% BSA, overnight, 4°C), rabbit anti–human SPTLC1 (Proteintech, 15376-1-AP; 1:1,000 in 5% BSA, overnight, 4°C), rabbit anti–human CerS6 (Invitrogen, Lass6; 1:1,000 in 5% BSA, overnight, 4°C), rabbit anti-TIP47/PLIN3 (Proteintech, 10694-1-AP; 1:1,000, overnight, 4°C), or mouse anti–β-actin IgG (MilliporeSigma, AC-74; 1:5,000, 2 hours, room temperature), followed by horseradish peroxidase–conjugated sheep anti-mouse IgG (Amersham, NA931; 1:10,000, 1 hour, room temperature) or horseradish peroxidase–conjugated donkey anti-rabbit IgG (Amersham, NA934; 1:10,000, 1 hour, room temperature). Band intensity was detected by chemiluminescence (Amersham), and images were generated using the Azure 300Q system. Immunoblots were conducted a minimum of 3 times, and one representative blot is shown in figures. Band intensities were quantified by ImageJ (NIH) and are shown in histogram scatterplots as mean ± SEM.

### ER length measurements.

Cells were fixed in 4% paraformaldehyde, and ERs were demarcated by immunocytochemical labeling with mouse Alexa Fluor 488–conjugated anti–human calnexin IgG (Santa Cruz Biotechnology, sc-23954; 1:25, overnight, 4°C). Images were obtained using an Olympus confocal microscope and saved as TIFF files. Tubular length of peripheral ER was then measured using the pixel-to-nm ratio conversion and free hand tracing tools within NIH ImageJ software. ER branches greater than 90° were considered separate organelles.

### Lipid droplet detection.

Kidney tissue frozen sections or cultured proximal tubule cells were fixed in 4% paraformaldehyde and permeabilized with 0.1% Triton X-100. In some experiments, LipidSpot 488 stain (Biotium, 70065) was applied (1:1,000, 30 minutes, room temperature). Images were viewed with an Olympus confocal microscope, at λ = 488 nm excitation and λ = 587 emission. In other experiments, lipid droplets (LDs) were detected with rabbit anti-TIP47/PLIN3 IgG (Proteintech; 1:200, 3 hours, room temperature) and Alexa Fluor 594–conjugated donkey anti-rabbit IgG (Invitrogen, A-21207; 1:200, 1 hour, room temperature), with λ = 590 nm excitation and λ = 618 nm emission.

### Ceramide concentration measurements.

Ceramides were extracted from kidney homogenates and cultured cells as previously described (88). For total kidney ceramides, approximately 25 mg of frozen tissue was homogenized in 300 μL buffer B (250 mM sucrose, 25 mM KCl, 50 mM Tris-base, 0.5 mM EDTA). Homogenates corresponding to 2.4 ± 0.21 mg total protein, along with calibration standards for assay linearity, were spiked with 50 μL of internal standard C18:1/17:0 (50 ng). Lipids were extracted using the Bligh and Dyer method (89), and the organic phase was dried under nitrogen gas and stored at –80°C until analysis. For ceramide extraction from cultured cells, pellets from about 10^6^ cells (~1 mg protein) were suspended in 0.5 mL of chloroform/methanol (1:2) in 13 × 100 mm screw-cap glass tubes. After addition of 30 μL of internal standard C18:1/17:0 (30 ng), lipids were extracted as described above. Both tissue and cell extracts were analyzed using the same LC-MS/MS method (88).

Ceramides were quantified by HPLC-MS/MS (Thermo Fisher Scientific) using electrospray ionization in positive ion mode and multiple reaction monitoring (MRM). Standards and samples were resuspended in 50 μL 0.1% formic acid in methanol/water (85:15) and injected into a Waters 2690 HPLC system equipped with a Vydac 200MS C8 column (2.1 × 100 mm, 5 μm; P.J. Cobert Associates). MRM transitions monitored the common fragment ion at *m*/*z* 264 for all ceramide species. Specific MRM transitions and retention times for individual ceramides are provided in Supplemental Table 1. Calibration curves used to assess the linearity of the assay included ceramide 18:1/14:0, 18:1/16:0, 18:1/18:0, 18:1/18:1, 18:1/20:0, 18:1/22:0, 18:1/24:0, and 18:1/24:1 using ceramide 18:1/17:0 internal standard. Quantification was performed using ceramide C18:1/17:0 as the internal standard, and data were analyzed with Xcalibur software (Thermo Fisher Scientific, v3.0.63).

### Ceramide transfection.

To test the direct effect of ceramides on ER stress, we initially incubated cells with C16:0 ceramide in ethanol/dodecane (98:2, vol/vol). Although this protocol has been used for introducing short-chain ceramides, we were unsuccessful with C16:0 ceramide (not shown). Thus, we developed a protocol to express long-chain ceramides by transfection. NBD–C12 ceramide or unlabeled C16:0 ceramide (Avanti Polar Lipids; 10 μM complexed with 0.4% albumin in Opti-MEM) was mixed 1:1 (vol/vol) with Lipofectamine 3000 (Thermo Fisher Scientific; 6% in Opti-MEM) and incubated for 15–20 minutes at room temperature. The complexed ceramide-Lipofectamine mixture was incubated with cells (2–4 hours, 37°C in humidified 5% CO_2_ incubator), washed with PBS, fixed with paraformaldehyde (4%, 5 minutes, room temperature), and permeabilized with Triton X-100 (0.15% in PBS, 20–30 minutes, room temperature). Cells were then rinsed with PBS, and blocked with donkey serum (30–60 minutes, room temperature), mounted with antifade medium, and shielded from light until microscopy studies were conducted. For NBD-ceramide experiments, excitation λ = 467 nm and emission λ = 538 nm.

### ER membrane fluidity measurements.

To assess ceramide-induced changes in organelle membrane fluidity, C-laurdan (Tocris Bioscience; 5 μM in phenol red–free PBS) was incubated with cells (30 minutes, 37°C in humidified 5% CO_2_ incubator), washed with PBS, and fixed with paraformaldehyde (4%, 5 minutes, room temperature). Cells were then rinsed with PBS and stored at 4°C until examination by confocal microscopy (excitation λ = 405 nm, emission λ = 440 nm in gel phase and 490 nm in liquid phase). ER membranes were labeled with LumiTracker ER Red (Lumiprobe; 1 μM, 30 minutes, 37°C, excitation λ = 589 nm, emission λ = 617 nm). ImageJ software was then used to calculate ratiometric C-laurdan fluorescence within a region of interest that is restricted to ER (61).

### Statistics.

Graphical data are presented as mean ± SEM and were analyzed using GraphPad Prism 7 software. Data from multiple groups were analyzed by 1-way ANOVA and Tukey’s post hoc test for multiple comparisons. Data from 2 groups were analyzed by unpaired 2-tailed *t* test. Statistical significance for all analyses was defined as a *P* value less than 0.05.

### Study approval.

All studies were conducted in accordance with protocols approved by the Institutional Animal Care and Use Committee of Case Western Reserve University School of Medicine.

### Data availability.

Data are available in the Supporting Data Values file. Raw RNA-seq data were generated by LC Sciences and are not available for posting to the NCBI’s Gene Expression Omnibus platform. Differential gene expression data were deposited at Zenodo (https://doi.org/10.5281/zenodo.20434667).

## Author contributions

ZL designed research studies, conducted experiments, acquired data, analyzed data, and reviewed/edited the manuscript. RJG, SK, VL, AC, US, and TK conducted experiments, acquired data, analyzed data, and reviewed/edited the manuscript. FJM and TM analyzed data and reviewed/edited the manuscript. JRS obtained funding, designed research studies, analyzed data, and wrote and edited the manuscript.

## Conflict of interest

The authors have declared that no conflict of interest exists.

## Funding support

This work is the result of NIH funding and is subject to the NIH Public Access Policy. Through acceptance of this federal funding, the NIH has been given a right to make the work publicly available in PubMed Central.

- NIH 5R01NS105789 and 1R21NS145426 to TM.
- NIH 2R01DK067528 to JRS.

## Supplementary Material

Supplemental data

Unedited blot and gel images

Supporting data values

## Version 1 06/18/2026

In-Press Preview

## Version 2 08/10/2026

Electronic publication
