# Supplementary material for: Apical proximal tubule fatty acid uptake–generated ceramides cause endoplasmic reticulum stress from altered membrane fluidity
Source: JCI Insight. 2026 Jun 18;11(15):e199699. doi: 10.1172/jci.insight.199699 (PMC13463616; doi:10.1172/jci.insight.199699)
Supplement: Supplementary file 1 [file jciinsight-11-199699-s010.pdf]

Supplemental Table 1. LC/MS transitions and retention times for all analyzed ceramide species.

Ceramide	Transition (m/z)	Retention time (minutes)
17:0/18:1	552.6 → 264	4.42
14:0/18:1	510.5 → 264	4.10
16:0/18:1	538.5 → 264	4.32
18:0/18:1	566.4 → 264	4.50
18:1/18:1	564.4 → 264	4.35
20:0/18:1	594.4 → 264	4.69
22:0/18:1	622.6 → 264	4.84
24:0/18:1	650.6 → 264	4.98
18:1/24:1	648.6 → 264	4.81

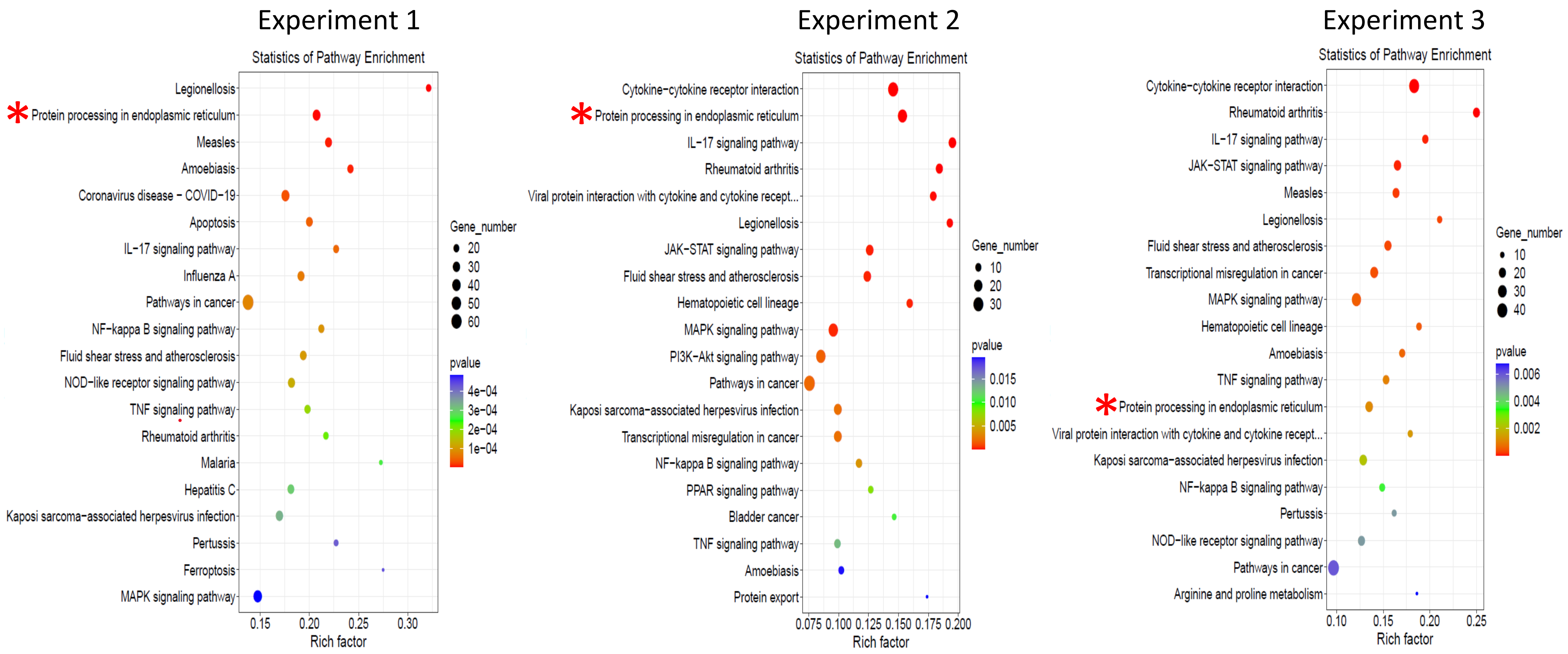

Supplemental Figure 1. AP + BL vs. BL palmitate RNAseq pathway analysis. Kyoto Encyclopedia of Genes and Genomes (KEGG) analyses from three separate experiments comparing transcript expression between human proximal tubule cells incubated with bilateral vs. basolateral palmitate (100 µM, 16 hr, 37° C). Bubble size is proportional to the number of annotated genes in the pathway. Rich factor = number of differentially expressed genes in the pathway/all genes in the pathway. Color corresponds to p-value. * denotes the pathway corresponding to ER stress.

Experiment 1 | Experiment 2 | Experiment 3

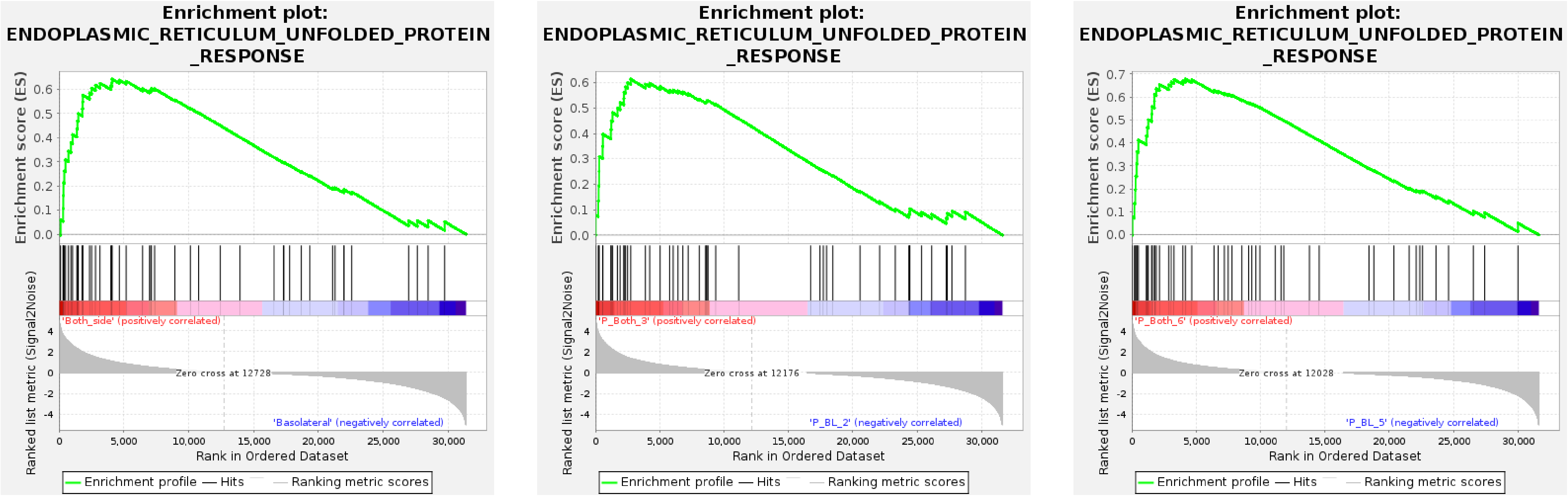

Experiment 1	Experiment 2	Experiment 3
ES = 0.64, NES = 2.06	ES = 0.61, NES = 1.96	ES = 0.68, NES = 2.08
FDR q-value = 7.5×10^{-3}	FDR q-value = 0.1	FDR q-value = 5.5×10^{-3}

Supplemental Figure 2. Gene set enrichment analysis (GSEA) for the unfolded protein response. GSEA from three separate experiments comparing transcript expression between human proximal tubule cells incubated with bilateral vs. basolateral palmitate (100 µM, 16 hr, 37° C). Abbreviations: ES, enrichment score; NES, normalized enrichment score; FDR, false discovery rate.

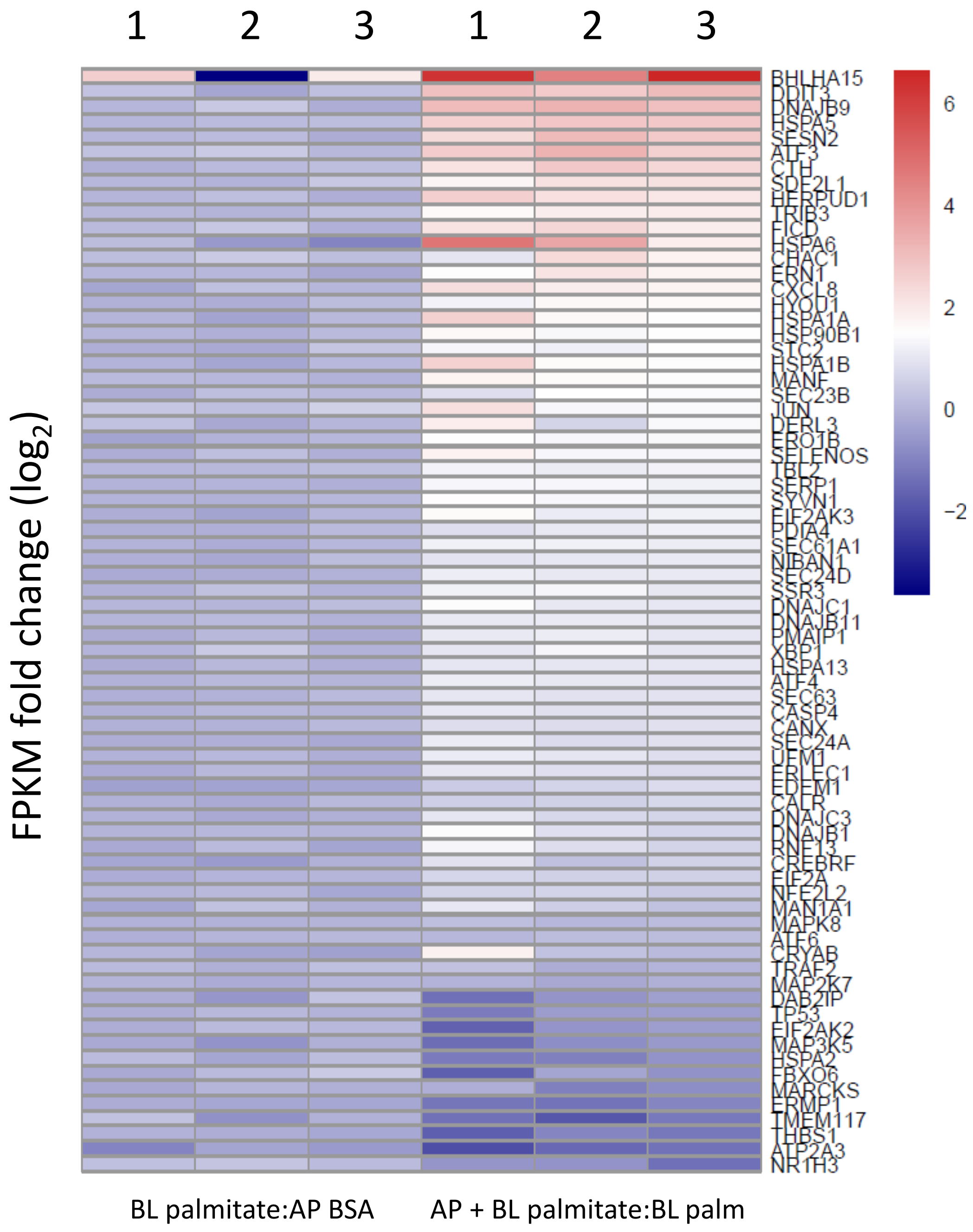

Supplemental Figure 3. Bilateral palmitate upregulates ER stress gene expression. Heat map from three separate experiments comparing transcript expression between human proximal tubule cells incubated with AP + BL vs. BL palmitate (100 µM complexed with 0.2% BSA, 16 hr, 37° C) in three right lanes, and three separate experiments comparing transcript expression between human proximal tubule cells incubated with BL palmitate complexed with (100 µM complexed with 0.2% albumin, 16 hr, 37° C) vs. AP albumin (0.2%, 16 hr, 37° C). Abbreviations: FPKM, fragments per kB transcript per million mapped reads.

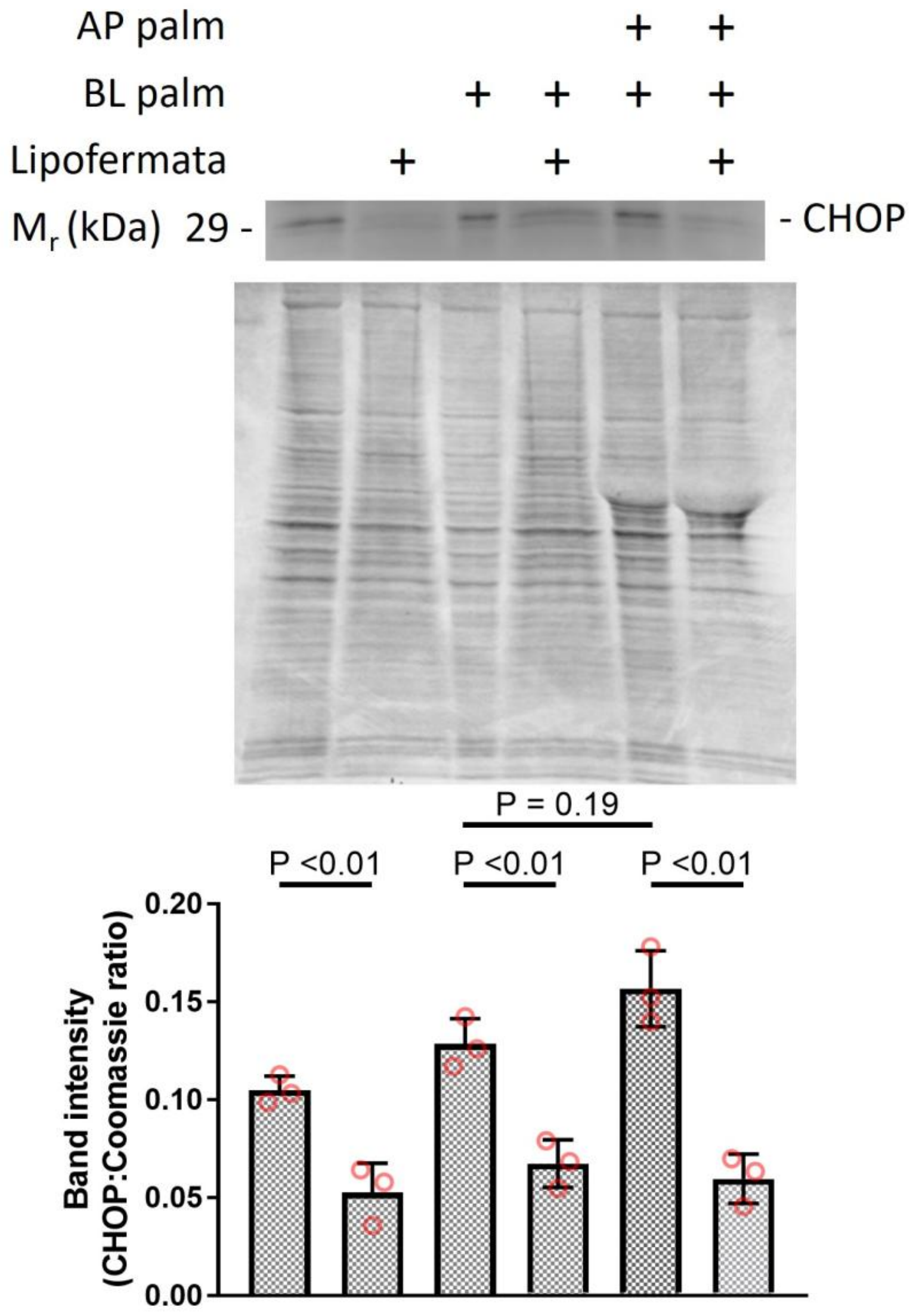

Supplemental Figure 4. Human proximal tubule cells were pre-treated with 25 µM Lipofermata ± BL or AP + BL palmitate (100 µM complexed with 0.2% BSA, 16 hr, 37° C). Lysate were immunoblotted for CHOP; gels were stained with Coomassie blue, and ratios of band:lane density was calculated using ImageJ. Data are means ± SEM from three experiments.

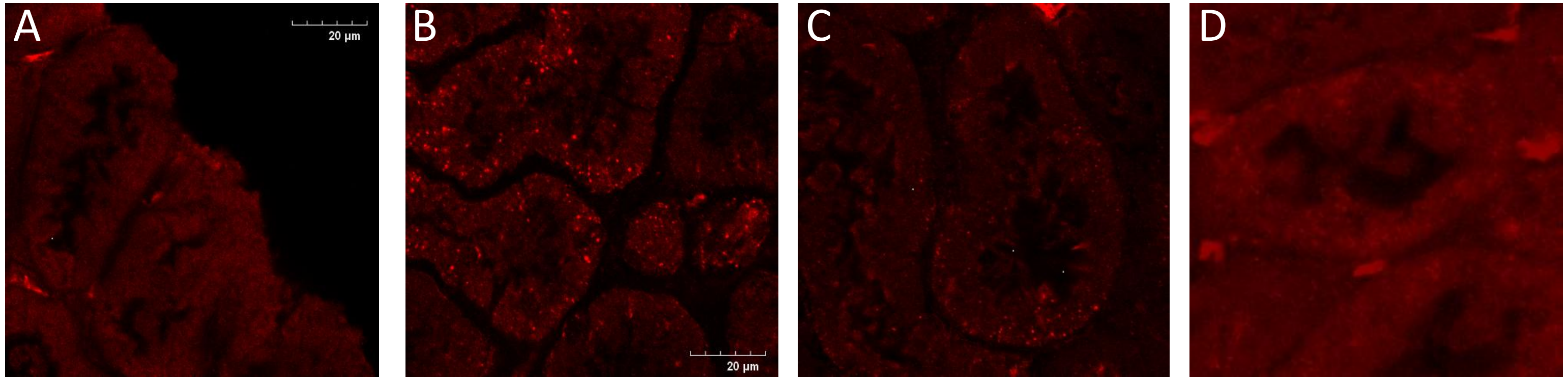

Supplemental Figure 5. Lipid droplets in mouse proximal tubules. Kidney frozen sections from wild-type (A), db/db (B), db/db *eNOS*$^{-/-}$ (C) and db/db *eNOS*$^{-/-}$ *Slc27a2*$^{-/-}$ (D) mice were stained for lipid droplets by immunohistochemistry using rabbit anti-PLIN3 and Texas red-conjugated anti-rabbit IgG as described in Methods.

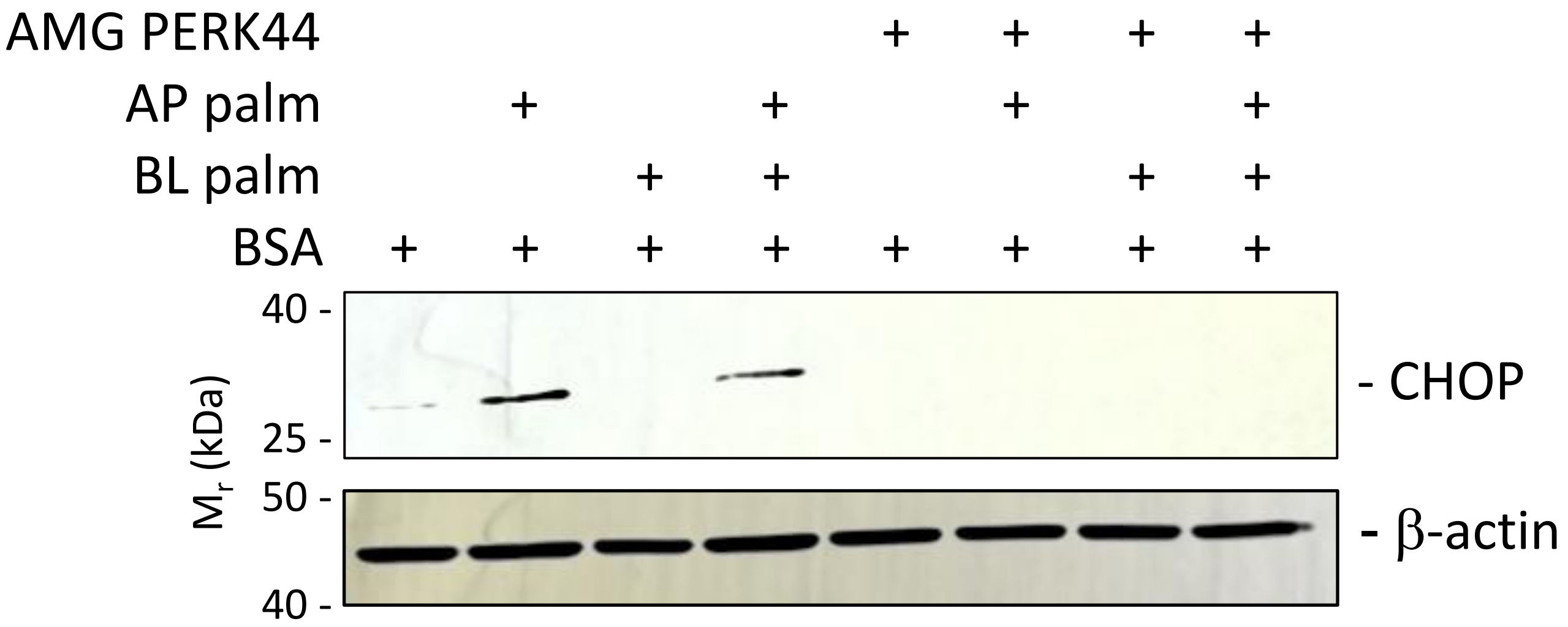

Supplemental Figure 6. AMG PERK44 inhibits ER stress.
Human proximal tubule cells were pre-treated with the PERK inhibitor AMG PERK44 (1 µM, 1 hr, 37° C), then with AP and/or BL palmitate (100 µM complexed with 0.2% BSA, 16 hr, 37° C). Cell lysates were probed for CHOP or β-actin by immunoblot analysis, as described in Methods.

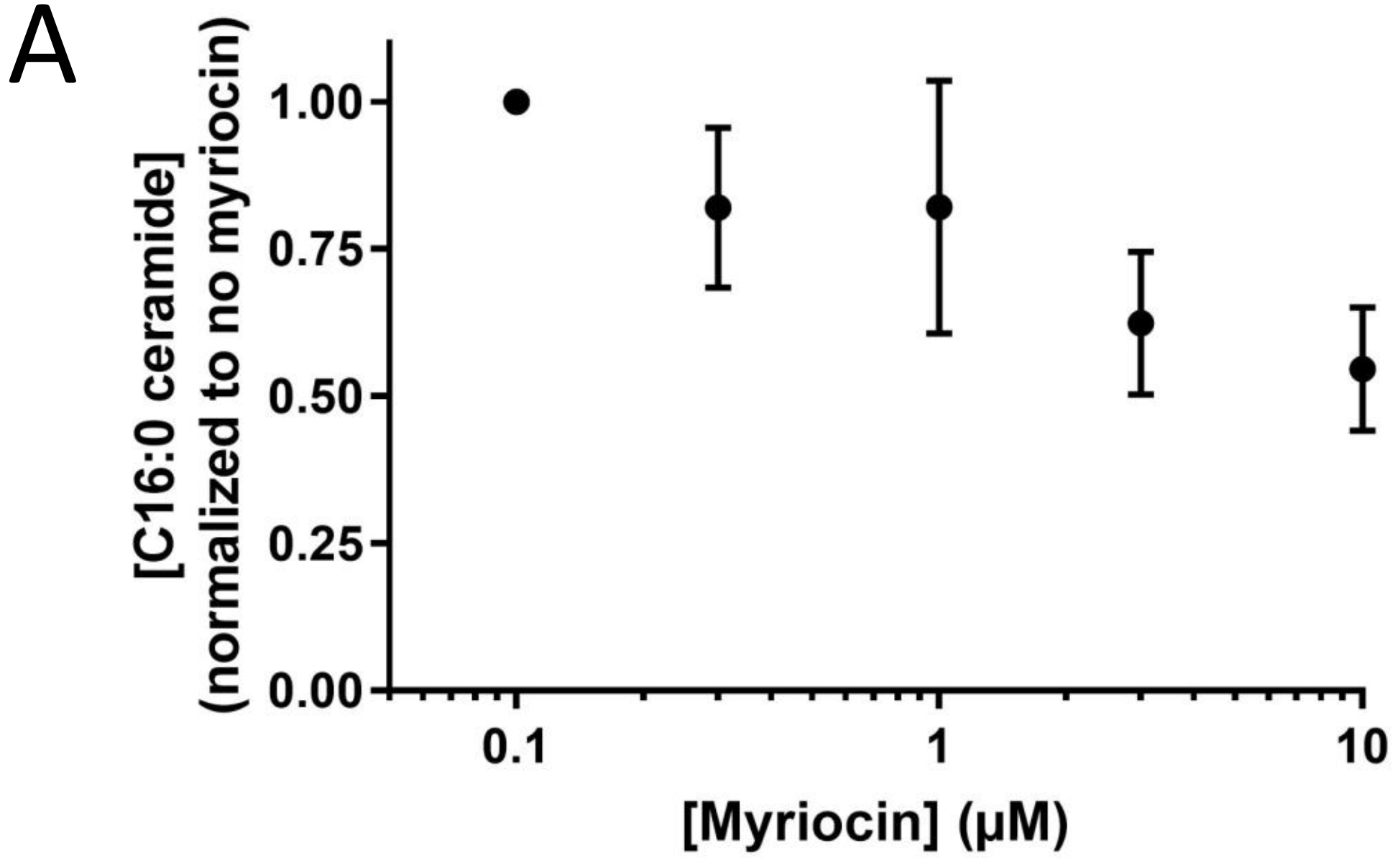

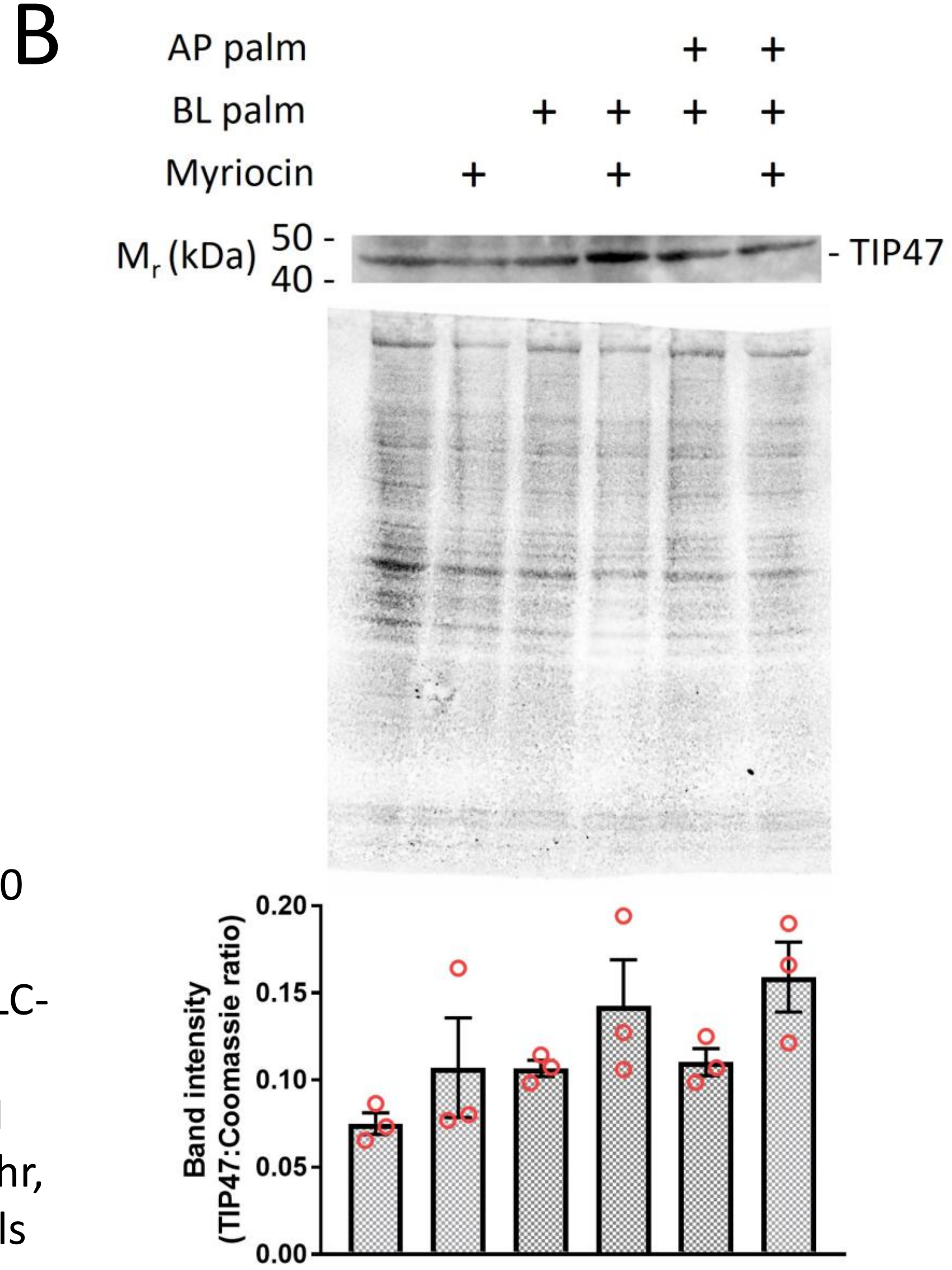

Supplemental Figure 7. Myriocin effects on ceramide synthesis and lipid droplet (LD) biogenesis. A. Human proximal tubule cells were pre-treated with myriocin at indicated concentrations, then with AP + BL palmitate (100 µM complexed with 0.2% BSA, 16 hr, 37° C). Cells were scraped from permeable supports into glass vials, frozen, and assayed for ceramides by LC-MS/MS as described in Methods. Data are means ± SEM from five experiments. B. Human proximal tubule cells were pre-treated with 10 µM myriocin ± BL or AP + BL palmitate (100 µM complexed with 0.2% BSA, 16 hr, 37° C). Lysates were immunoblotted for LDs with anti-TIP47 antibodies; gels were stained with Coomassie blue, and ratios of band:lane density were calculated using ImageJ. Data are means ± SEM from three experiments.

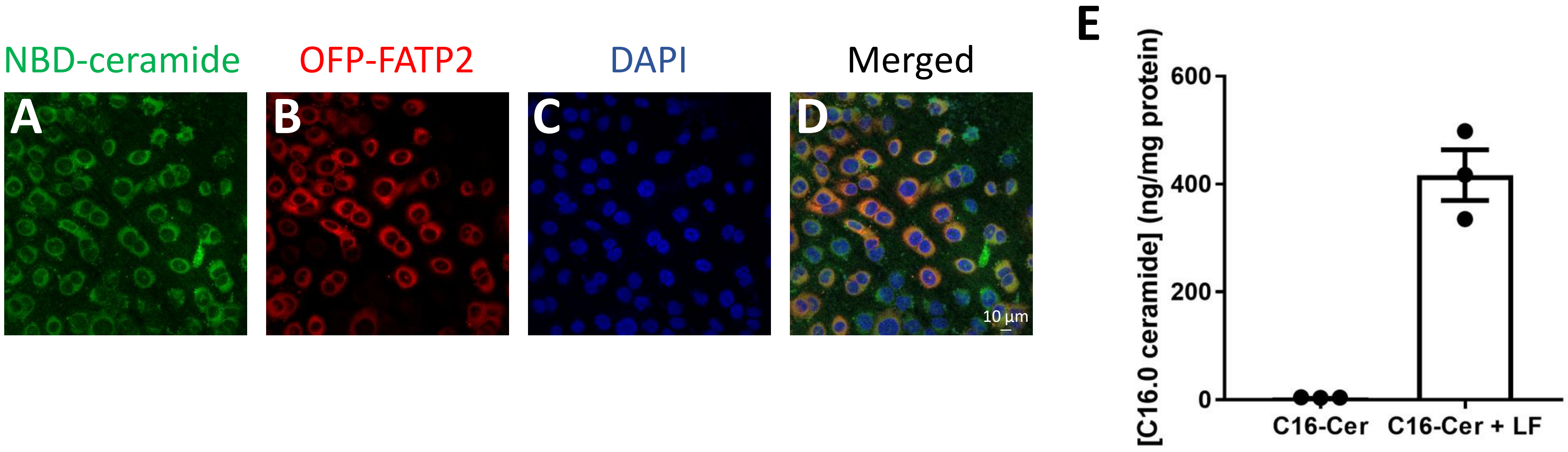

Supplemental Figure 8. Introduction of ceramide by lipofection. (A-D) Stable OFP-tagged FATP2-expressing human proximal tubule cells on permeable supports were incubated with NBD-tagged C12-ceramide complexed with Lipofectamine 3000 (as described in Methods). Cells were fixed in paraformaldehyde, mounted with DAPI-containing media, and viewed by confocal microscopy. (E) Human proximal tubule cells on permeable supports were incubated with C16:0 ceramide complexed with Lipofectamine 3000 or vehicle (2-4 hrs, 37° C in humidified 5% CO_2 incubator). Cells were scraped from permeable supports into glass vials, frozen, and assayed for ceramides by LC-MS/MS as described in Methods. Data are means ± SEM from three experiments.

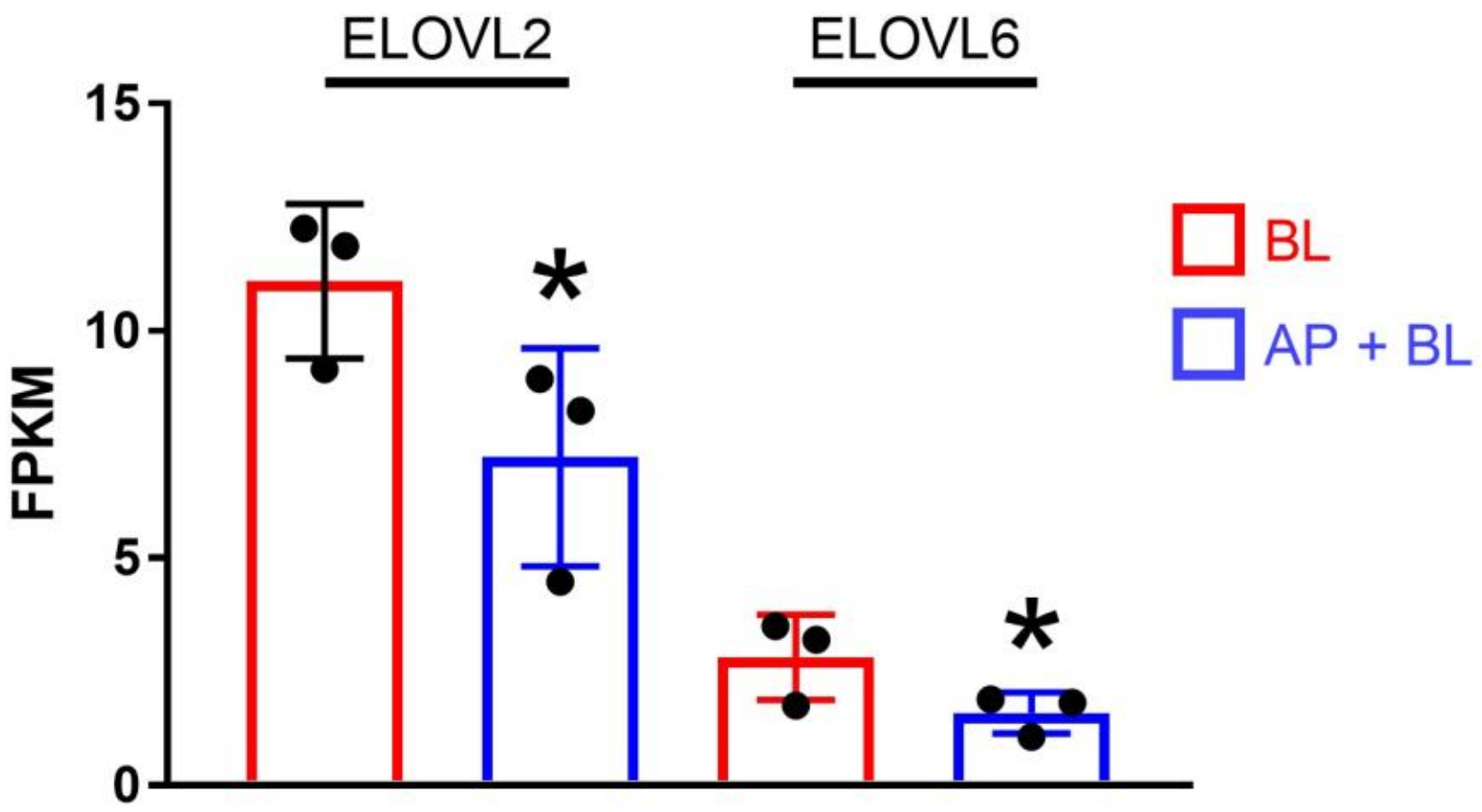

Supplemental Figure 9. Expression of long-chain fatty acid elongase mRNAs. Long-chain fatty acid elongase gene transcript expression in human proximal tubule cells treated with AP + BL vs. BL palmitate (100 µM complexed with 0.2% BSA, 16 hr, 37° C). Data are mean ± SD. * P <0.05 compared to BL palmitate group by paired t-test. Abbreviations: ELOVL2, fatty acid elongase 2; ELOVL6, fatty acid elongase 6; FPKM, fragments per kB transcript per million mapped reads.
