# Supplementary material for: Apical proximal tubule fatty acid uptake–generated ceramides cause endoplasmic reticulum stress from altered membrane fluidity
Source: JCI Insight. 2026 Jun 18;11(15):e199699. doi: 10.1172/jci.insight.199699 (PMC13463616; doi:10.1172/jci.insight.199699)

Figure 3A

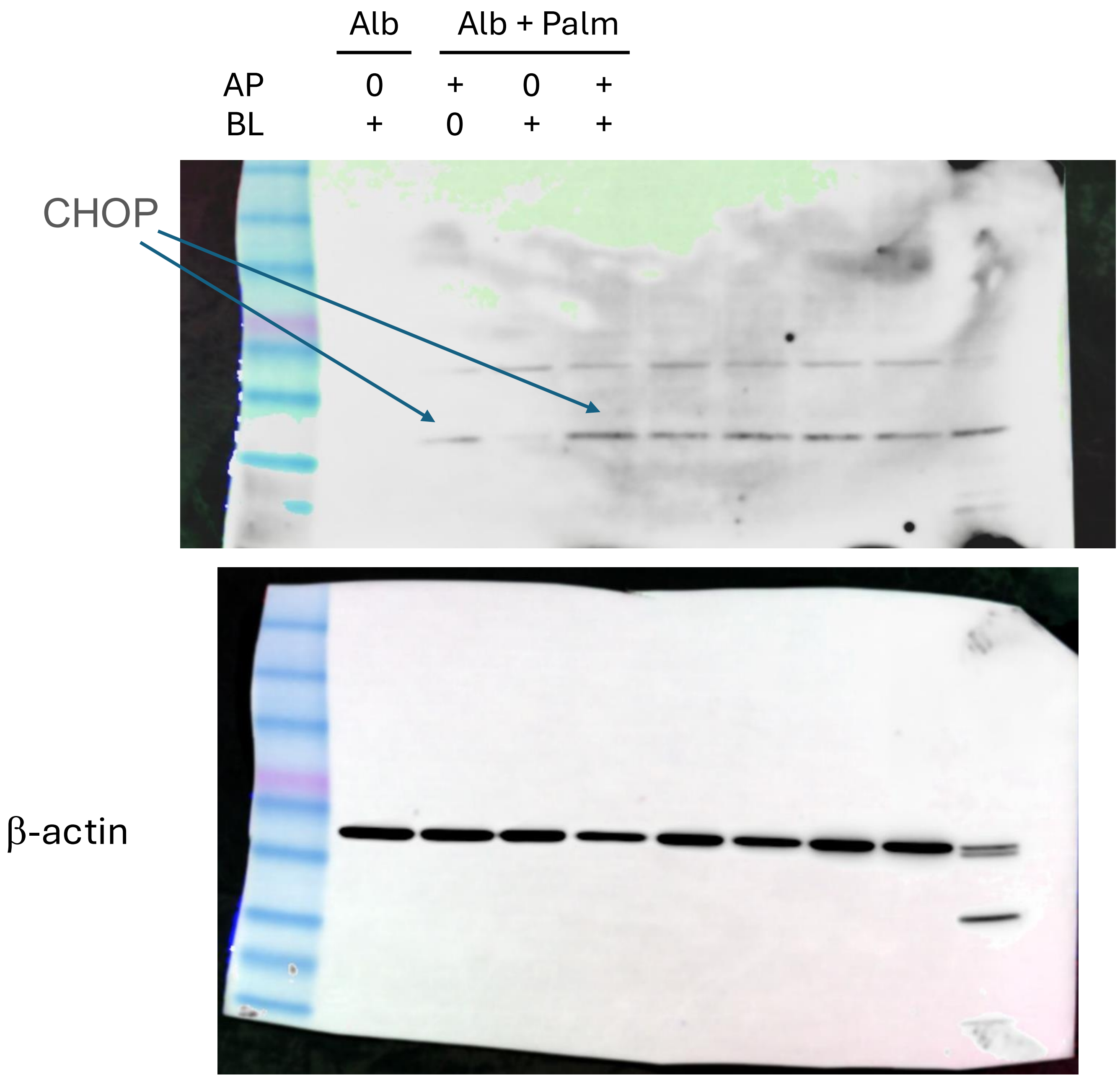

Figure 3C

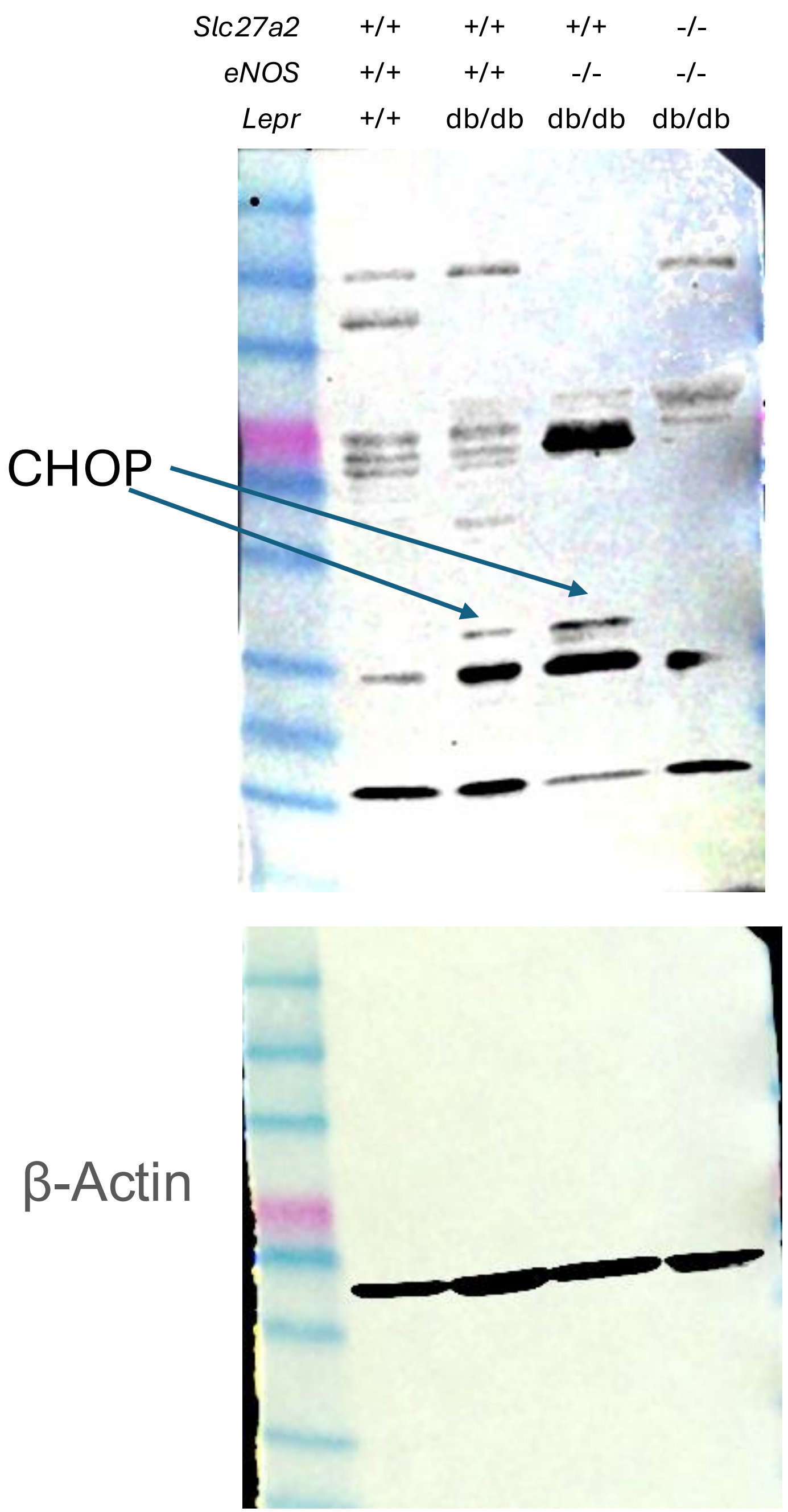

Supplemental Figure 4

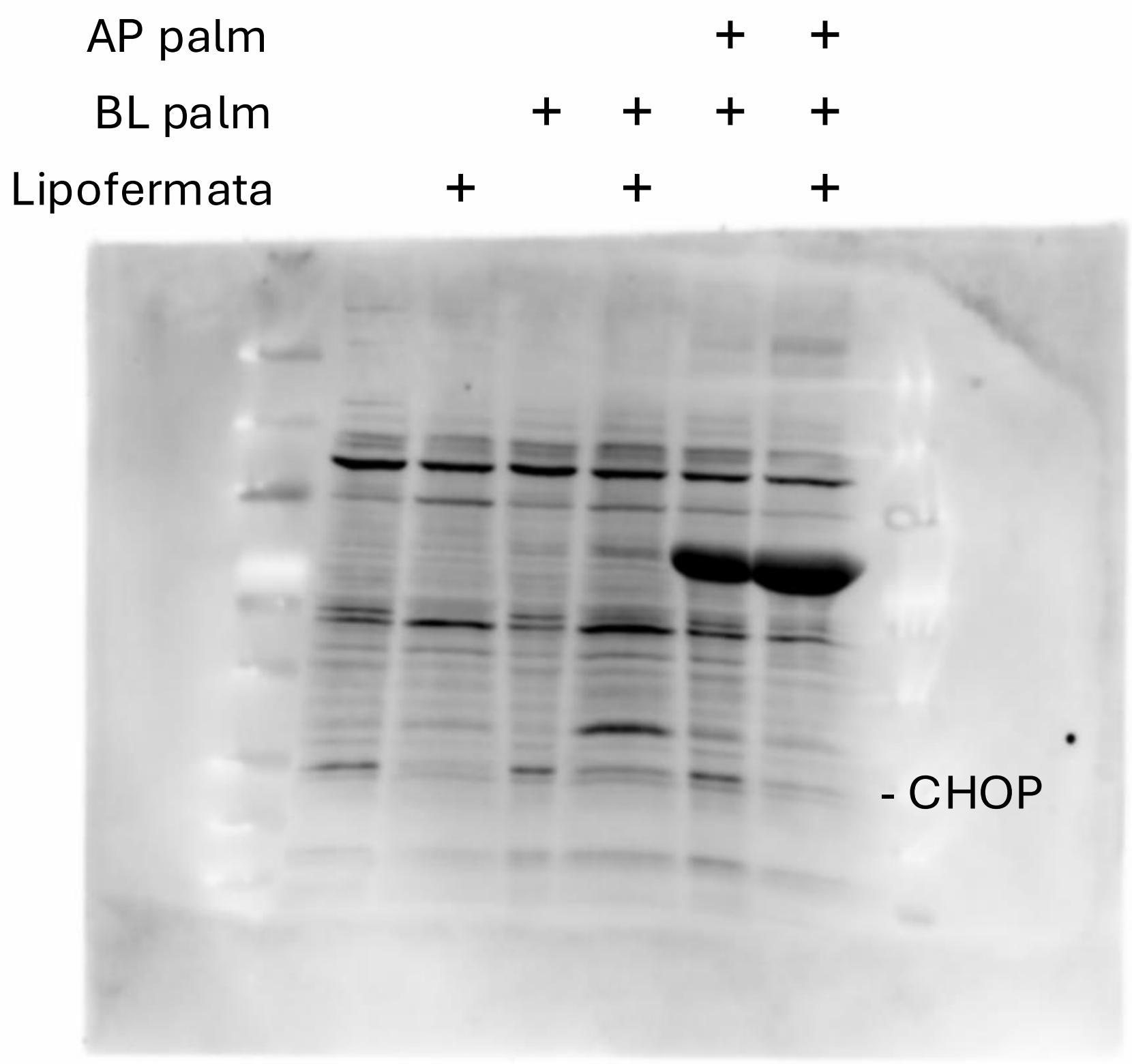

Figure 5E

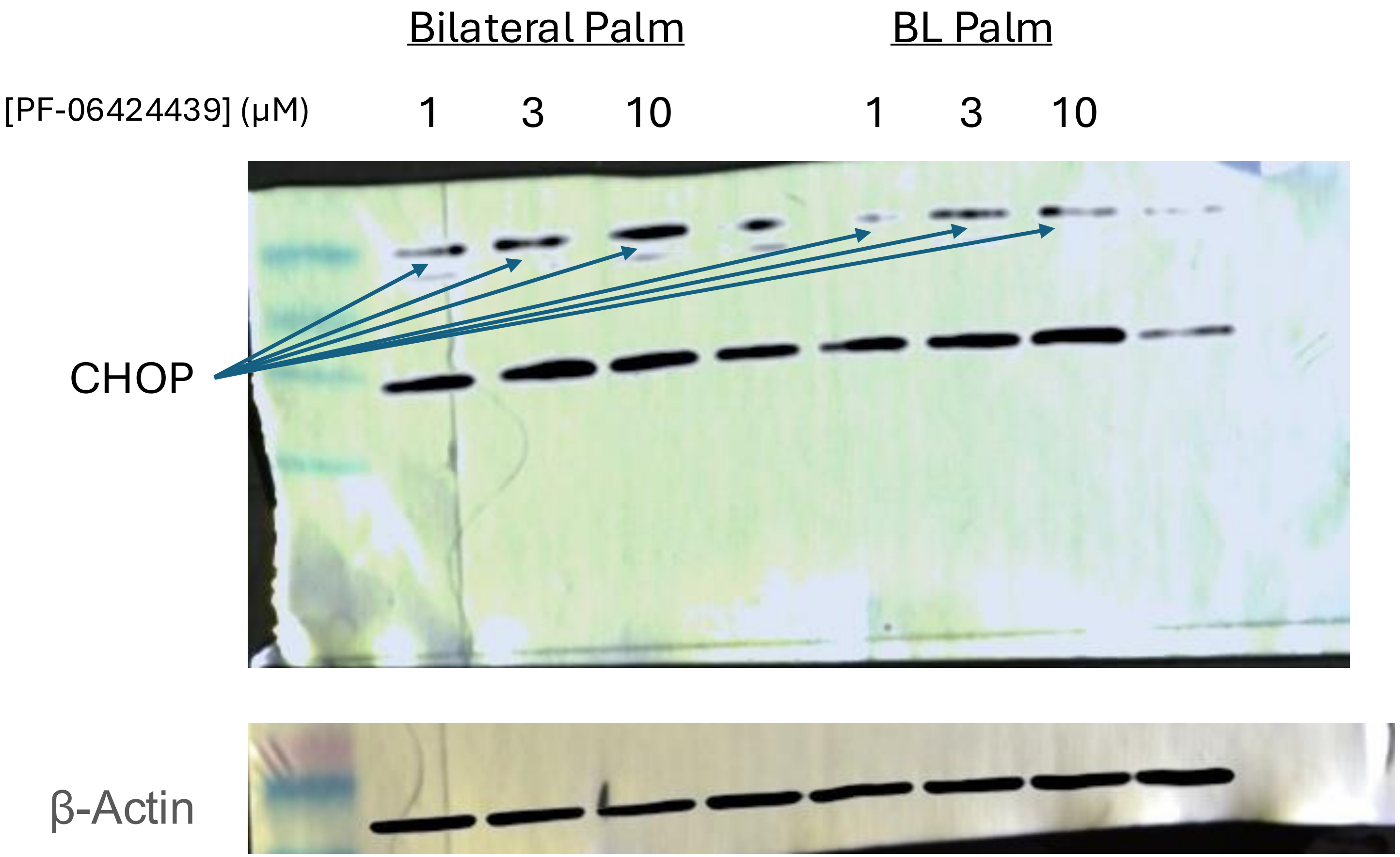

Figure 7G

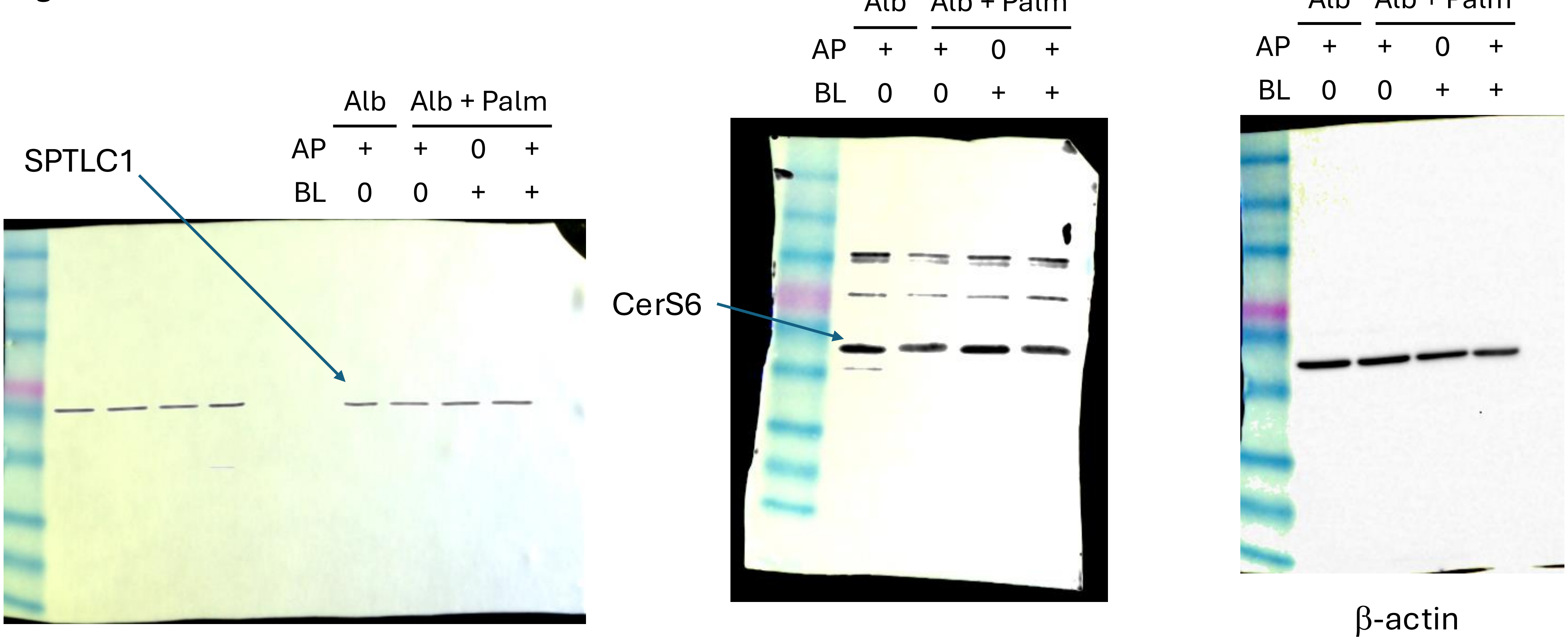

SPTLC1
Alb
Alb + Palm
AP + + 0 +
BL 0 0 + +
CerS6
Alb
Alb + Palm
AP + + 0 +
BL 0 0 + +
Alb
Alb + Palm
AP + + 0 +
BL 0 0 + +
β-actin

Supplemental Figure 6

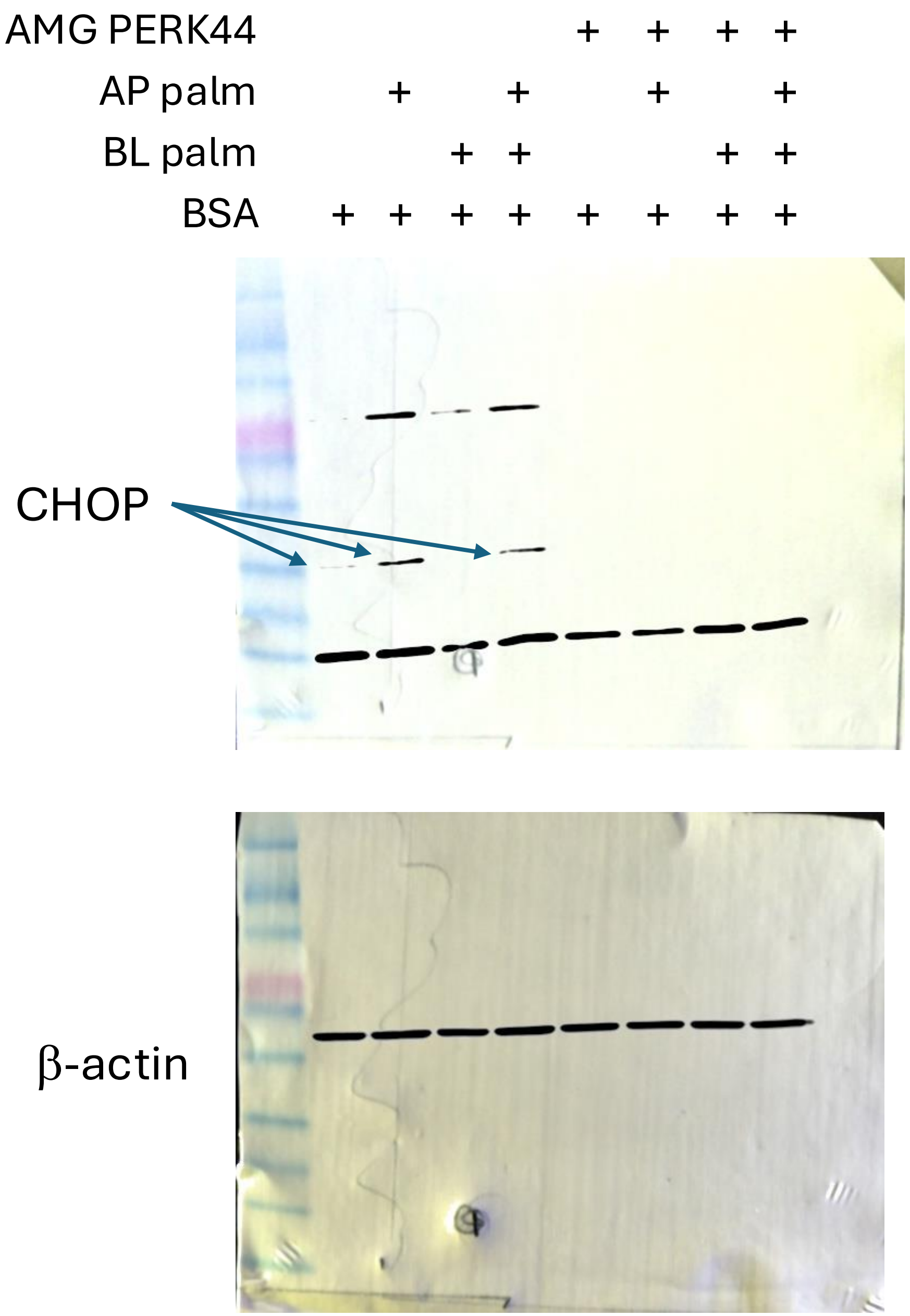

Figure 8B

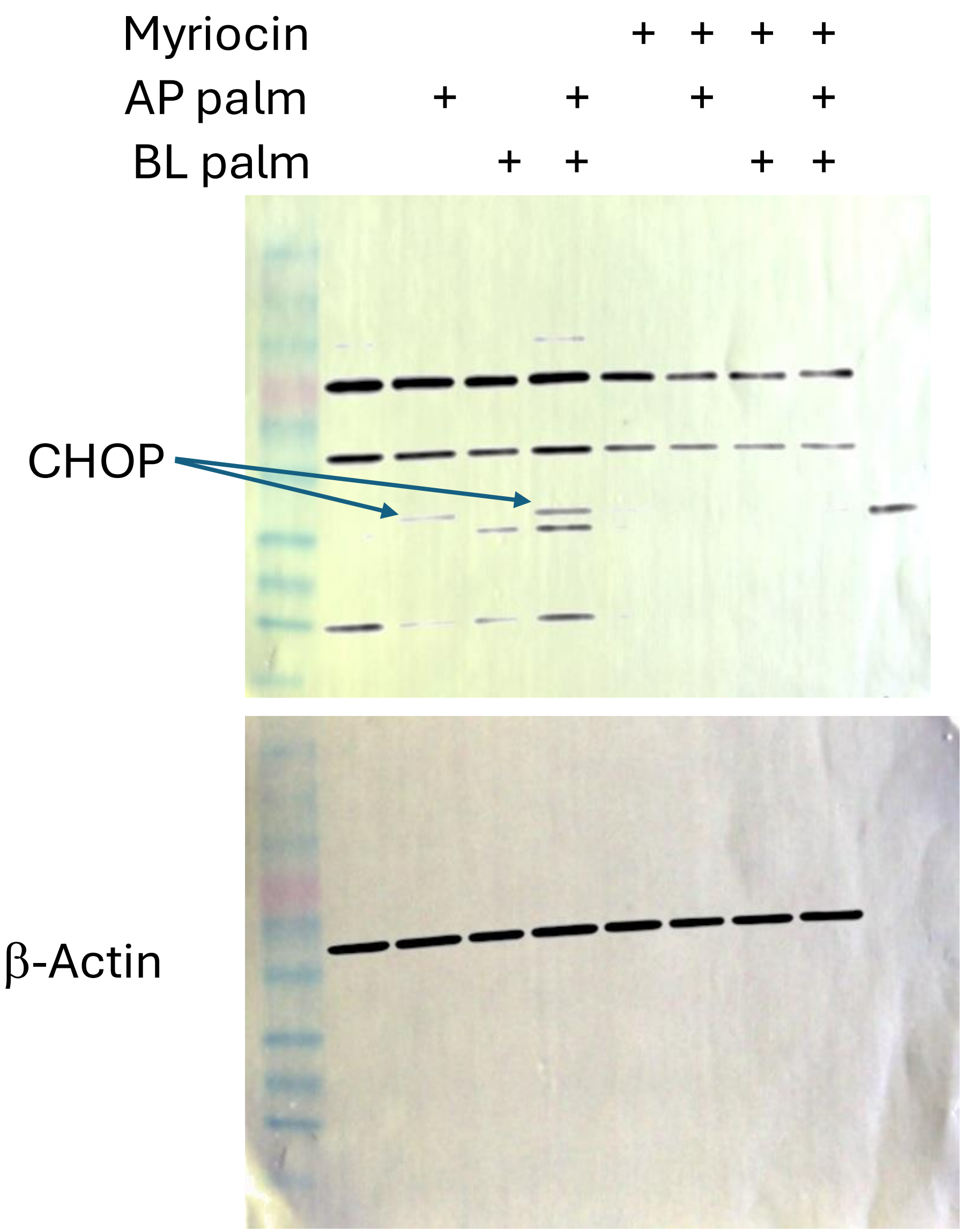

Figure 8C

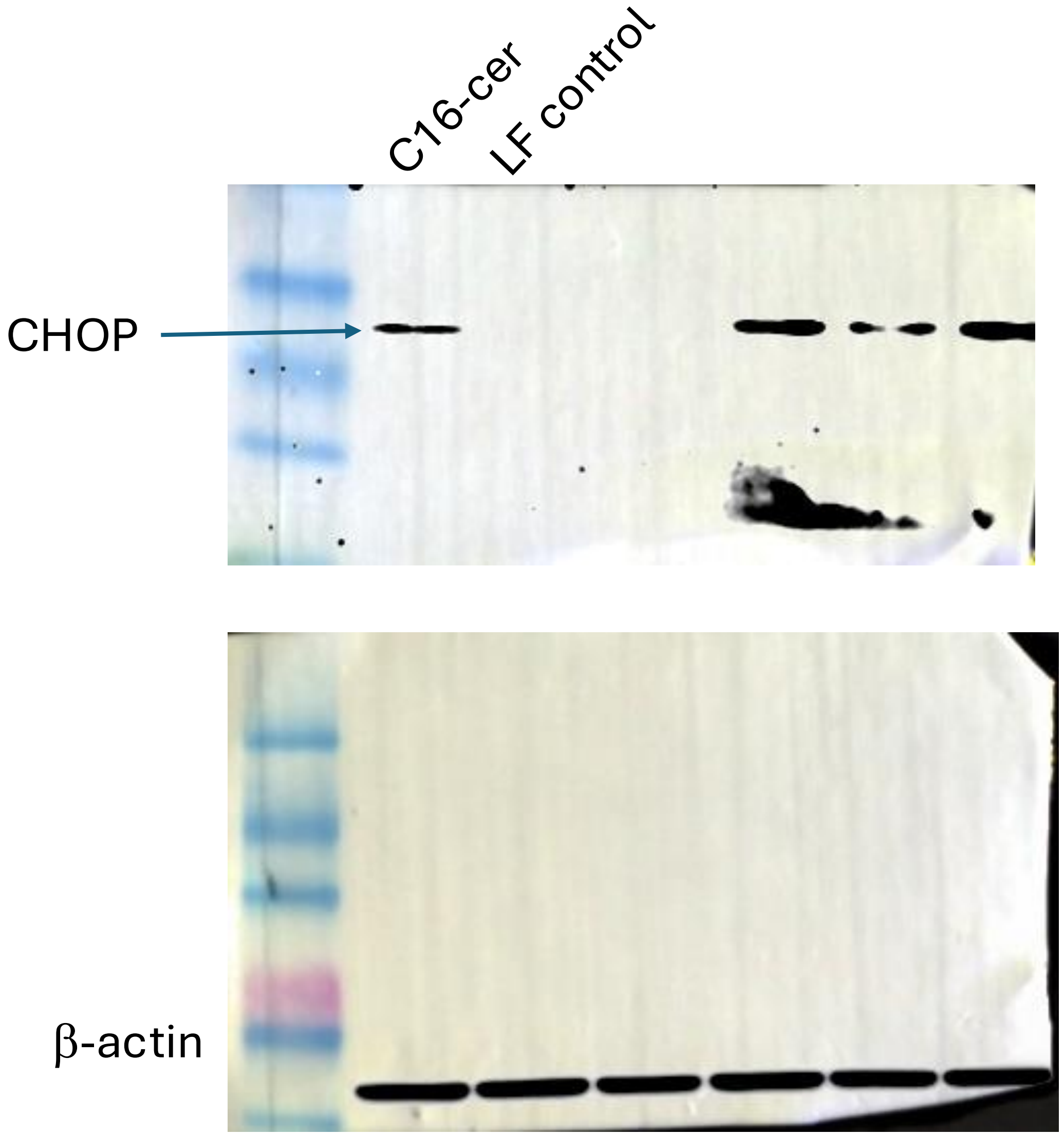

Supplemental Figure 7B

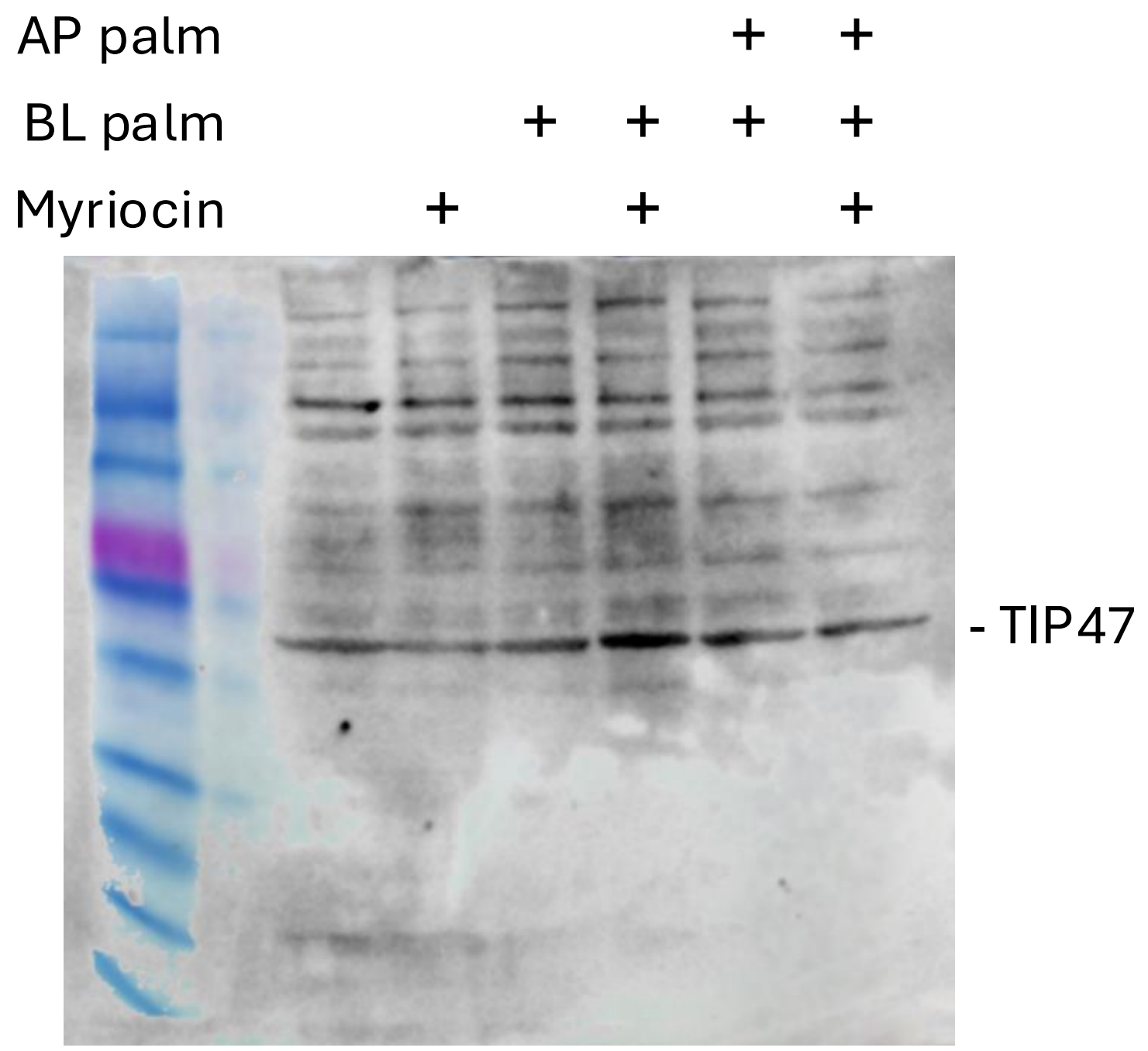

Supplement: Supplementary file 2 [file jciinsight-11-199699-s011.pdf]
